# Inhibition of forward and reverse transport of Ca^2+^ via Na^+^/Ca^2+^ exchangers (NCX) prevents sperm capacitation

**DOI:** 10.1186/s40659-024-00535-9

**Published:** 2024-08-23

**Authors:** Marc Yeste, Adeel Ahmad, Estel Viñolas, Sandra Recuero, Sergi Bonet, Elisabeth Pinart

**Affiliations:** 1 Biotechnology of Animal and Human Reproduction (TechnoSperm), Institute of Food and Agricultural Technology, University of Girona, Girona, ES-17003 Spain; 2 Unit of Cell Biology, Department of Biology, Faculty of Sciences, University of Girona, Girona, ES-17003 Spain; 3 Catalan Institution for Research and Advanced Studies (ICREA), Barcelona, ES-08010 Spain

**Keywords:** Sperm, Na^+^/Ca^2+^ exchangers (NCXs), In vitro capacitation and Ca^2+^ transport

## Abstract

**Background:**

While calcium is known to play a crucial role in mammalian sperm physiology, how it flows in and out of the male gamete is not completely understood. Herein, we investigated the involvement of Na^+^/Ca^2+^ exchangers (NCX) in mammalian sperm capacitation. Using the pig as an animal model, we first confirmed the presence of NCX1 and NCX2 isoforms in the sperm midpiece. Next, we partially or totally blocked Ca^2+^ outflux (forward transport) via NCX1/NCX2 with different concentrations of SEA0400 (2-[4-[(2,5-difluorophenyl)methoxy]phenoxy]-5-ethoxyaniline; 0, 0.5, 5 and 50 µM) and Ca^2+^ influx (reverse transport) with SN6 (ethyl 2-[[4-[(4-nitrophenyl)methoxy]phenyl]methyl]-1,3-thiazolidine-4-carboxylate; 0, 0.3, 3 or 30 µM). Sperm were incubated under capacitating conditions for 180 min; after 120 min, progesterone was added to induce the acrosome reaction. At 0, 60, 120, 130, and 180 min, sperm motility, membrane lipid disorder, acrosome integrity, mitochondrial membrane potential (MMP), tyrosine phosphorylation of sperm proteins, and intracellular levels of Ca^2+^, reactive oxygen species (ROS) and superoxides were evaluated.

**Results:**

Partial and complete blockage of Ca^2+^ outflux and influx via NCX induced a significant reduction of sperm motility after progesterone addition. Early alterations on sperm kinematics were also observed, the effects being more obvious in totally blocked than in partially blocked samples. Decreased sperm motility and kinematics were related to both defective tyrosine phosphorylation and mitochondrial activity, the latter being associated to diminished MMP and ROS levels. As NCX blockage did not affect the lipid disorder of plasma membrane, the impaired acrosome integrity could result from reduced tyrosine phosphorylation.

**Conclusions:**

Inhibition of outflux and influx of Ca^2+^ triggered similar effects, thus indicating that both forward and reverse Ca^2+^ transport through NCX exchangers are essential for sperm capacitation.

**Supplementary Information:**

The online version contains supplementary material available at 10.1186/s40659-024-00535-9.

## Background

Calcium (Ca^2+^) plays a crucial role in sperm differentiation and epididymal maturation, and is needed for motility activation upon ejaculation, and for sperm capacitation in the female genital tract [1, 2]. The physiological relevance of this ion is supported by the great diversity of Ca^2+^ channels present in the sperm plasma membrane of both invertebrates and vertebrates [3, 4]. In mammals, CatSper has been reported to play a pivotal function during sperm capacitation, as it favors the sustained Ca^2+^ entrance that is required for motility hyperactivation and acrosome reaction before oocyte fertilization [1, 2, 5]. Yet, there are other transporters implicated in the Ca^2+^ influx in capacitated sperm, such as voltage-gated (CaV), cyclic nucleotide-gated (CNG), and transient-receptor potential (TRP) channels (reviewed in Delgado-Bermúdez et al. [2]). Furthermore, few studies suggested that Ca^2+^ efflux through Ca^2+^-ATPase (PMCA) and Na^+^/Ca^2+^ exchangers (NCX) could regulate sperm chemotaxis and capacitation [1, 3, 4, 6]. In rodents, it has been hypothesized that Ca^2+^ clearance avoids premature motility hyperactivation and acrosome exocytosis in capacitated sperm [1, 7]. All this evidence suggests that NCX may be relevant to sperm physiology in mammals.

Na^+^/Ca^2+^ exchangers, which belong to the solute transporter family SLC8A and the superfamily of Ca^2+^/cation antiporters (CaCA), are involved in the regulation of Ca^2+^ homeostasis [8–11]. In eukaryotic cells, NCX are expressed as dimers in the plasma membrane, and specific variants are found in the inner mitochondrial (NCLX variant [12]) and nuclear membranes (specific NCX1 variants [13]). While NCX usually export Ca^2+^ (forward mode) using the electrochemical gradient of Na^+^ at a ratio of 3 Na^+^:1 Ca^2+^ [9, 14–17], they can also favor Ca^2+^ influx and Na^+^ efflux at high intracellular Na^+^ levels (reverse mode) [18]. In mammals, three different NCX isoforms have been identified, NCX1, NCX2 and NCX3, each being coded by a specific gene (*SLC8A1-SLC8A3*) [8–10, 19–21]. NCX1 is present in nearly all mammalian cells, notwithstanding variants related to the cell type and tissue have been described [8–10, 16, 19, 21]. NCX2 localizes mainly in the brain, spinal cord, and gastrointestinal and renal systems, whereas NCX3 is confined to the brain and skeletal muscle [9, 10, 15, 22]. NCX monomers contain ten transmembrane helices (TM1-TM10) and a cytoplasmic loop (f-loop) between TM5 and TM6 [11, 14]. The loop has two adjacent regulatory domains of Ca^2+^ binding (CBD1 and CBD2), forming a head-tail tandem linked by a short connector [23–27]. These domains consist of seven antiparallel β-sheets and have four (CBD1) or two (CBD2) Ca^2+^ binding sites that are situated at one end [24]. The binding of four Ca^2+^ to the CBD1 domain leads to a conformational change resulting in NCX activation [28]. In contrast, the CBD2 domain has low affinity for Ca^2+^, so that binding only occurs when CBD1 is saturated [23, 24, 27]. Interactions between CBD1 and CBD2 are essential for NCX regulation [24]; it is, however, noticeable that mitochondrial NCLX monomers do not have CBD domains [15, 17, 29]. The N-terminus of the cytoplasmic loop contains an inhibitory peptide region (XIP domain) with only 20 amino acid residues, which accounts for the slow inactivation of the channel by intracellular Na^+^ [28, 30, 31]. Intracellular Ca^2+^ and Na^+^ levels have, therefore, an antagonistic role in regulating NCX activation and deactivation [24]. Finally, α1 and α2 repeats between TM2-TM3 and TM7-TM8, respectively, contain essential residues for Ca^2+^ binding and transport [30]. Remarkably, NCX have a wide distribution across cells and tissues, and possess a similar molecular structure.

To the best of our knowledge, the types of NCX isoforms expressed in the male and female reproductive tracts are unknown. In addition, and focusing on the male gamete, only few studies conducted in humans and hamsters demonstrated that the specific blockage of these exchangers is associated to alterations in sperm motility after ejaculation and during capacitation [3, 6, 32]. Yet, no previous research has addressed whether inhibiting these exchangers prevents mammalian sperm to capacitate and undergo the acrosome reaction induced by progesterone. In this study, we aimed to identify and localize the different NCX isoforms (NCX1, NCX2 and NCX3) in the plasma membrane of porcine sperm, and to determine their physiological relevance for capacitation and acrosome reaction. Identification and localization of these exchangers were conducted through immunoblotting and immunofluorescence, respectively. Following this, two specific NCX-blockers were used to inhibit partially or completely the forward transport (2-[4-[(2,5-difluorophenyl)methoxy]phenoxy]-5-ethoxyaniline, SEA0400 [33, 34]), and the reverse transport of Ca^2+^ (ethyl 2-[[4-[(4-nitrophenyl)methoxy]phenyl]methyl]-1,3-thiazolidine-4-carboxylate, SN-6 [35, 36]). Our results support that both forward and reverse transport of Ca^2+^ via NCX exchangers is essential for sperm capacitation.

## Results

### NCX1 and NCX2, but not NCX3, are present in porcine sperm

**Fig. 1:**
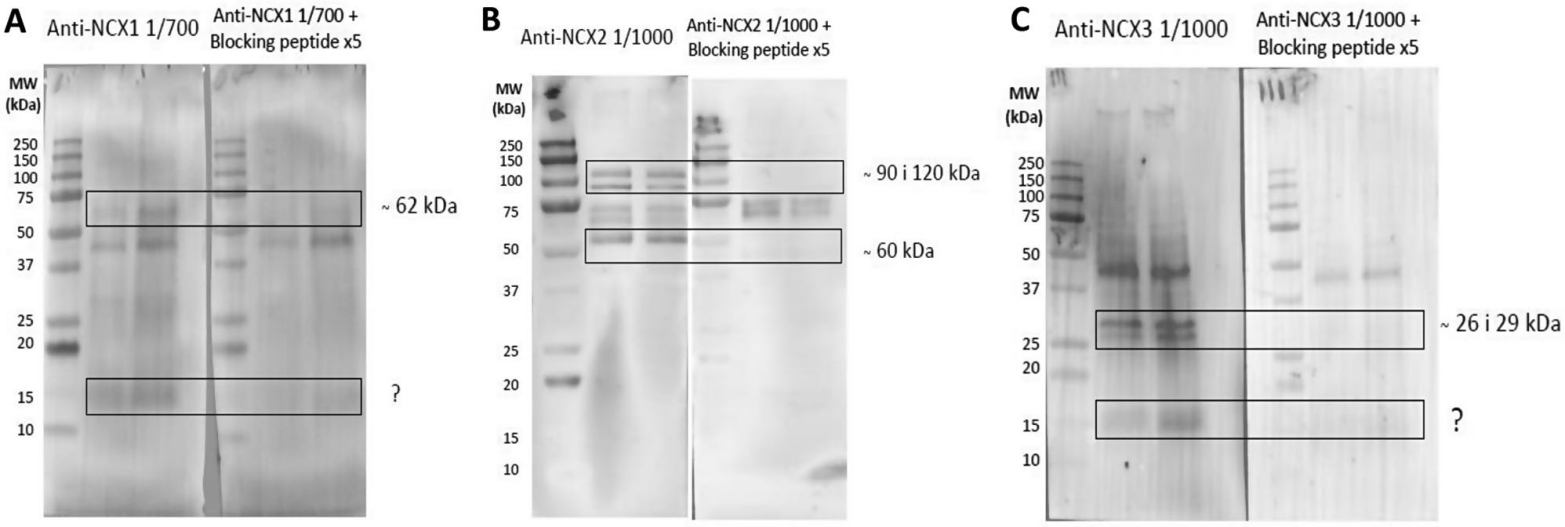
Identification of NCX isoforms. Identification of NCX1, NCX2 and NCX3 in porcine sperm. Representative immunoblots using sperm samples from different boars with **A**) anti-NCX1, (**B**) anti-NCX2 and (**C**) anti-NCX3 antibodies, and their respective blocking peptides

Immunoblotting assays revealed, for the first time, the presence of NCX1 (a 62-kDa), NCX2 (bands from 60 to 120 kDa) and NCX3 (< 30 kDa bands) in porcine sperm (Fig. 1A, B and C). Peptide blocking assays confirmed the specificity of primary antibodies.

**Fig. 2:**
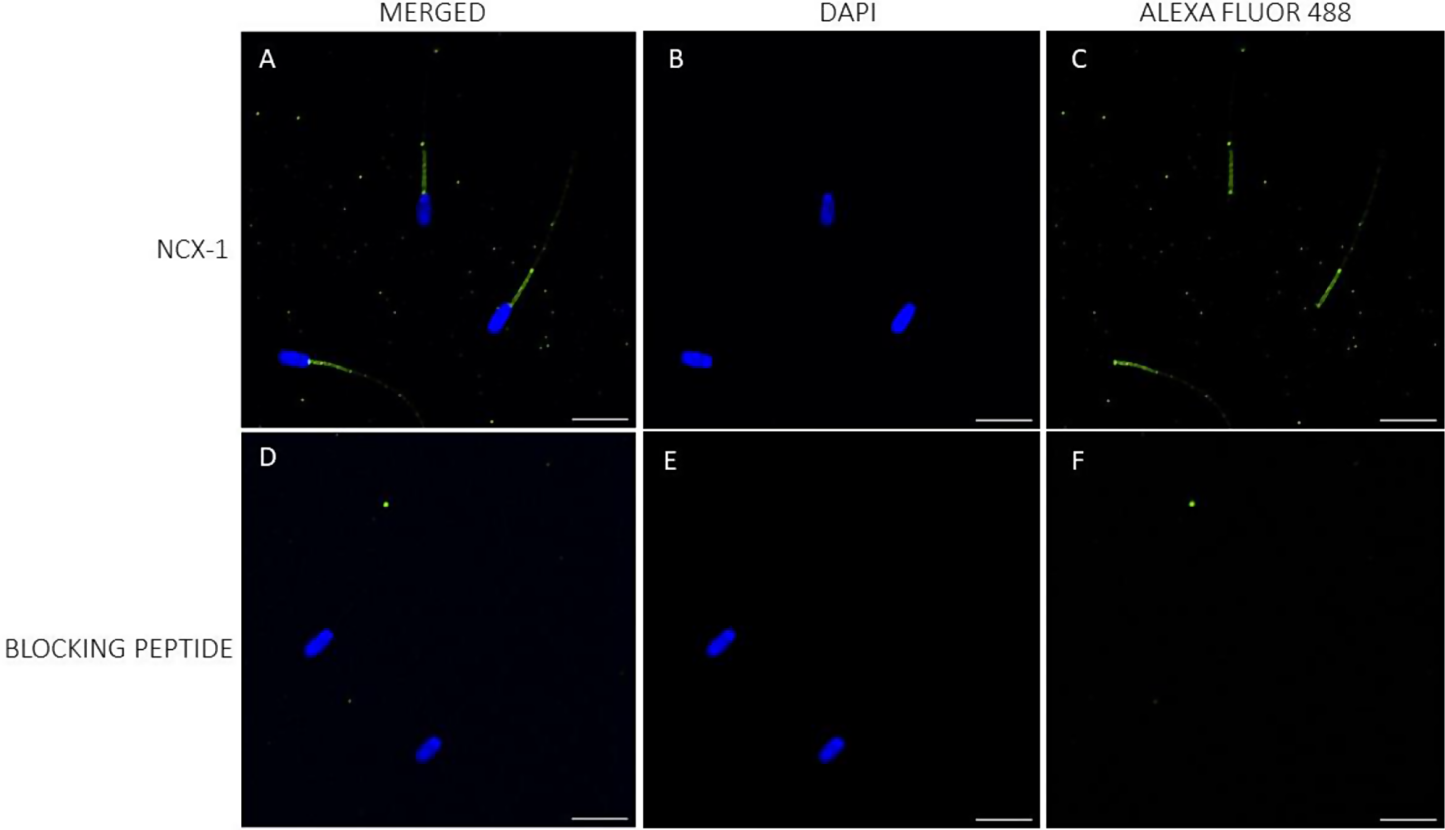
Immunolocalization of NCX1 isoform. Localization of NCX1 in the plasma membrane of porcine sperm (**A**–**C**), and after the peptide competition assay (**D**–**F**). NCX1 appears stained in green (Alexa Fluor 488) and nuclei in blue (DAPI; 4′6′-diamidion-2-phenylindole). Scale bar: 15 μm

**Fig. 3:**
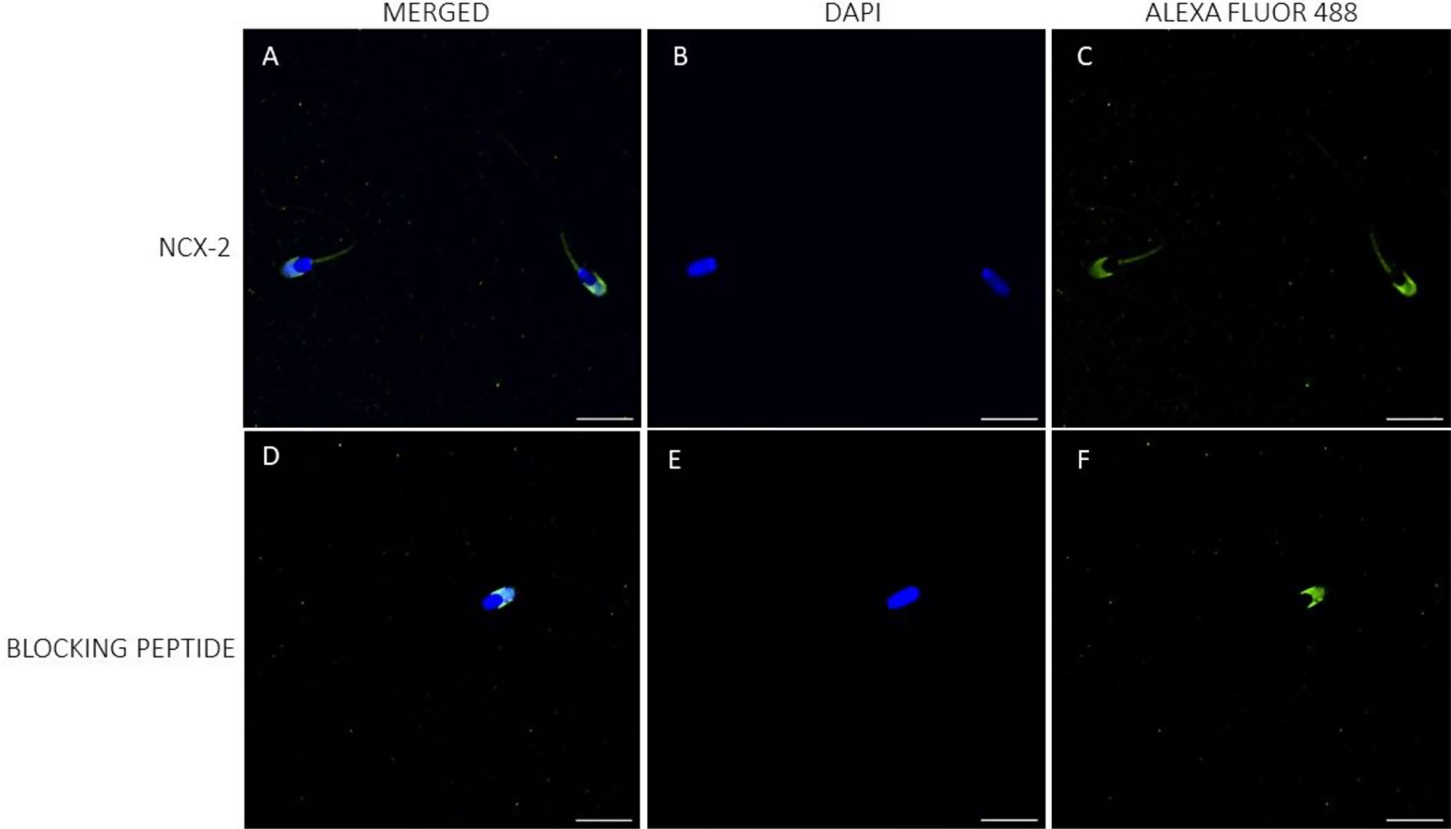
Immunolocalization of NCX2 isoform. Localization of NCX2 in the plasma membrane of porcine sperm (**A**–**C**), and after the peptide competition assay (**D**–**F**). NCX2 appears stained in green (Alexa Fluor 488) and nuclei in blue (DAPI; 4′6′-diamidion-2-phenylindole). Scale bar: 15 μm

**Fig. 4:**
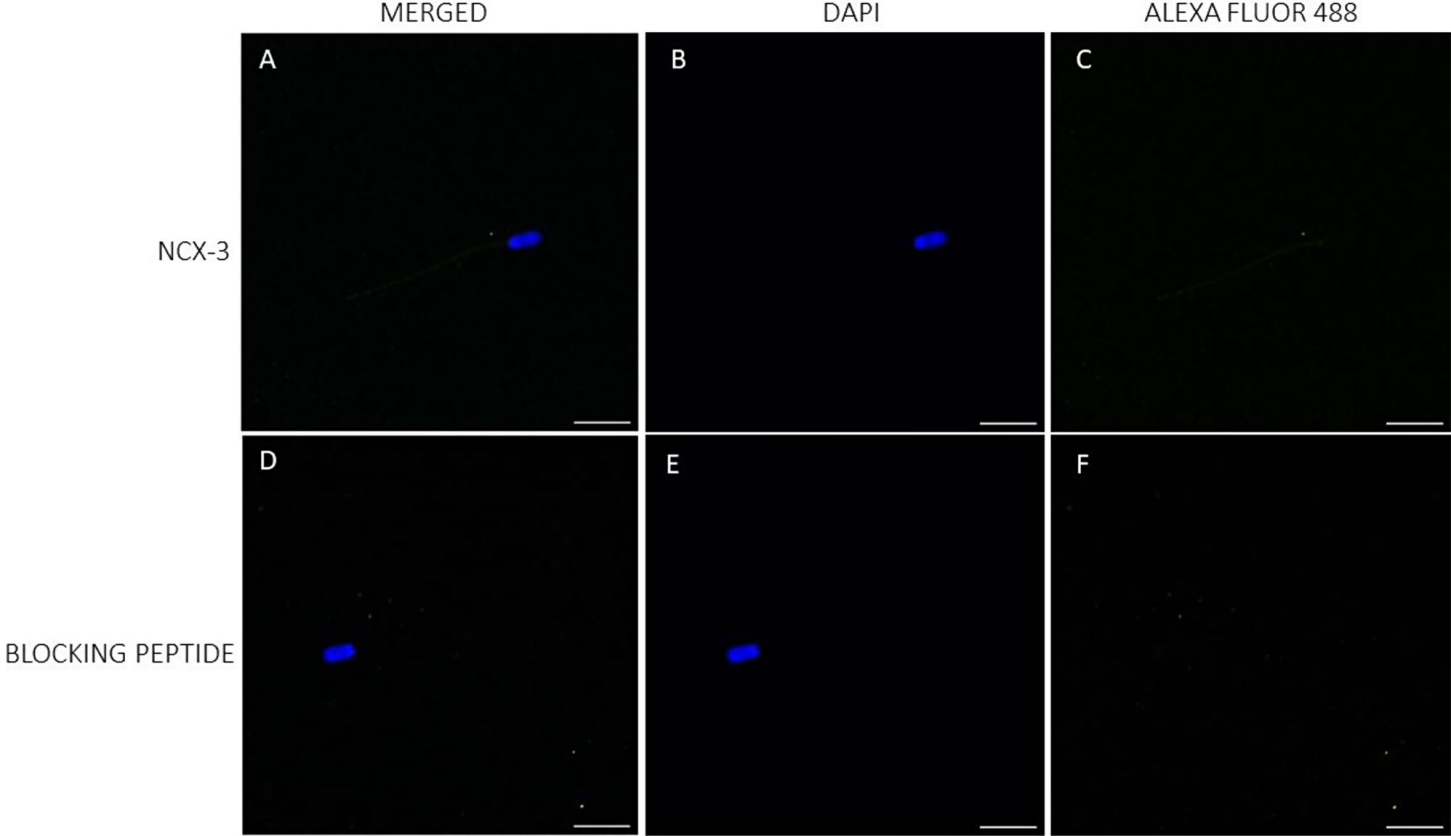
Immunolocalization of NCX3 isoform. Localization of NCX3 in the plasma membrane of porcine sperm (**A**–**C**), and after the peptide competition assay (**D**–**F**). NCX3 appears stained in green (Alexa Fluor 488) and nuclei in blue (DAPI; 4′6′-diamidion-2-phenylindole). Scale bar: 15 μm

While immunolocalization could verify the presence of NCX1 and NCX2 in porcine sperm, it failed to corroborate that of NCX3. In effect, NCX1 was found to be located in the connecting piece and midpiece (Fig. 2). In the case of NCX2, while intense staining in the acrosomal region and weak labeling in the midpiece were observed, peptide competition assays indicated that only the latter was specific (Fig. 3; Suppl. Fig. 1). Conversely, NCX3 could not be detected, which left immunoblotting results unconfirmed (Fig. 4).

### Blocking of NCX abolishes the transient increase in sperm motility induced by progesterone

**Fig. 5:**
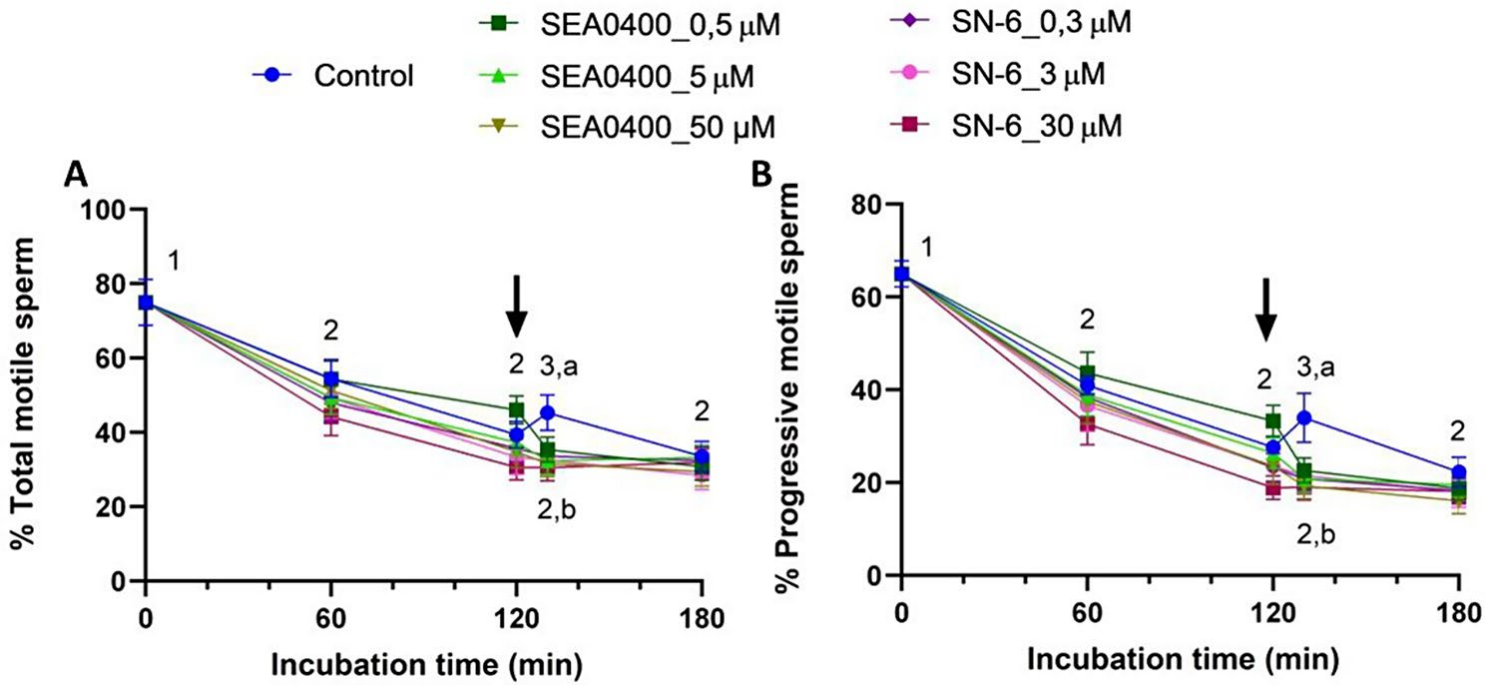
Sperm motility. Percentages of total (**A**) and progressively (**B**) motile sperm during in vitro capacitation of control samples and samples blocked with either SEA0400 (0.5, 5, and 50 µM) or SN-6 (0.3, 3, and 30 µM). Different superscript letters indicate significant differences between control and blocked samples within a single time point (*P* < 0.05). Different numeral superscripts indicate significant differences between time points within a treatment (*P* < 0.05). The arrow indicates the addition of 10 µg/mL of progesterone at 120 min of incubation. Results are expressed as the mean ± SEM (*n* = 10)

Total and progressive sperm motility continuously decreased during the first 120 min of incubation in all sperm samples, the differences between 0 and 120 min being statistically significant for both parameters (*P* < 0.001; Fig. 5). The addition of progesterone induced a significant but transient increase of total and progressive motility at 130 min in control samples (*P* < 0.001). Remarkably, this increase in motility was not observed in treated samples, regardless of blocker (SEA0400 or SN-6) and concentration (*P* > 0.05).

### Complete inhibition of NCX with SEA0400 or SN-6 decreases sperm velocity and ALH

**Fig. 6:**
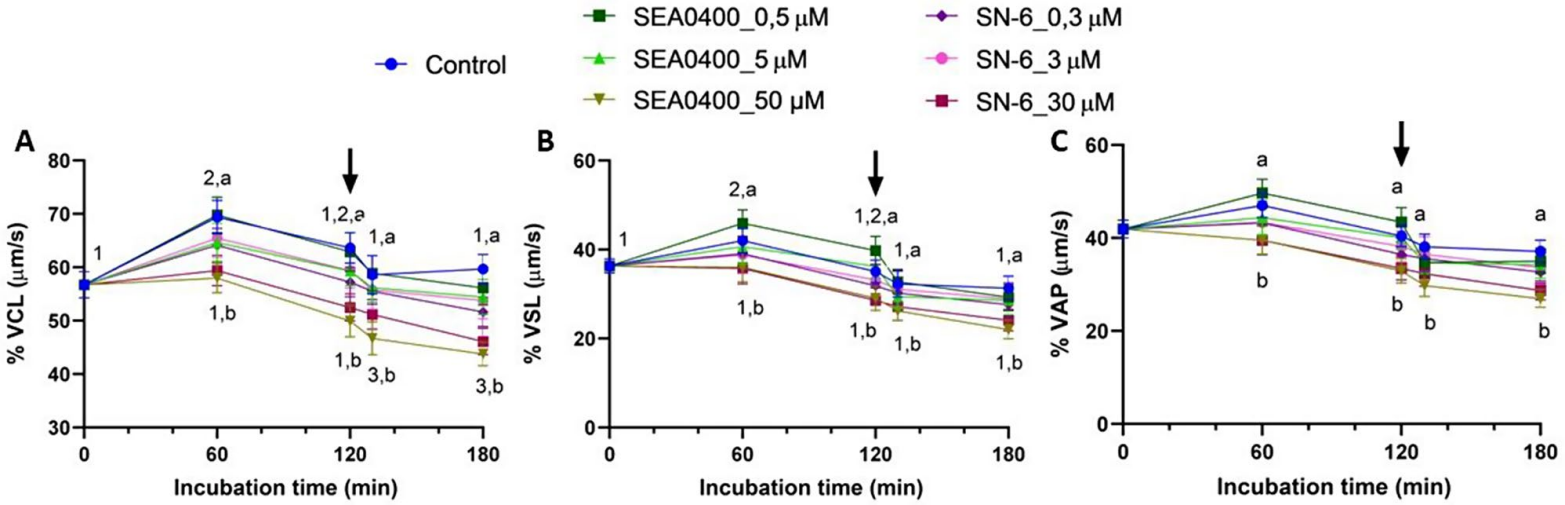
Sperm kinematics (I). Sperm velocity parameters of VCL (**A**), VSL (**B**), and VAP (**C**) during in vitro capacitation of control samples and samples blocked with either SEA0400 (0.5, 5, and 50 µM) or SN-6 (0.3, 3, and 30 µM). Different superscript letters indicate significant differences between control and blocked samples within a single time point (*P* < 0.05). Different superscript numbers indicate significant differences between time points within a treatment (*P* < 0.05). The arrow indicates the addition of 10 µg/mL of progesterone at 120 min of incubation. Results are expressed as the mean ± SEM (*n* = 10)

**Fig. 7:**
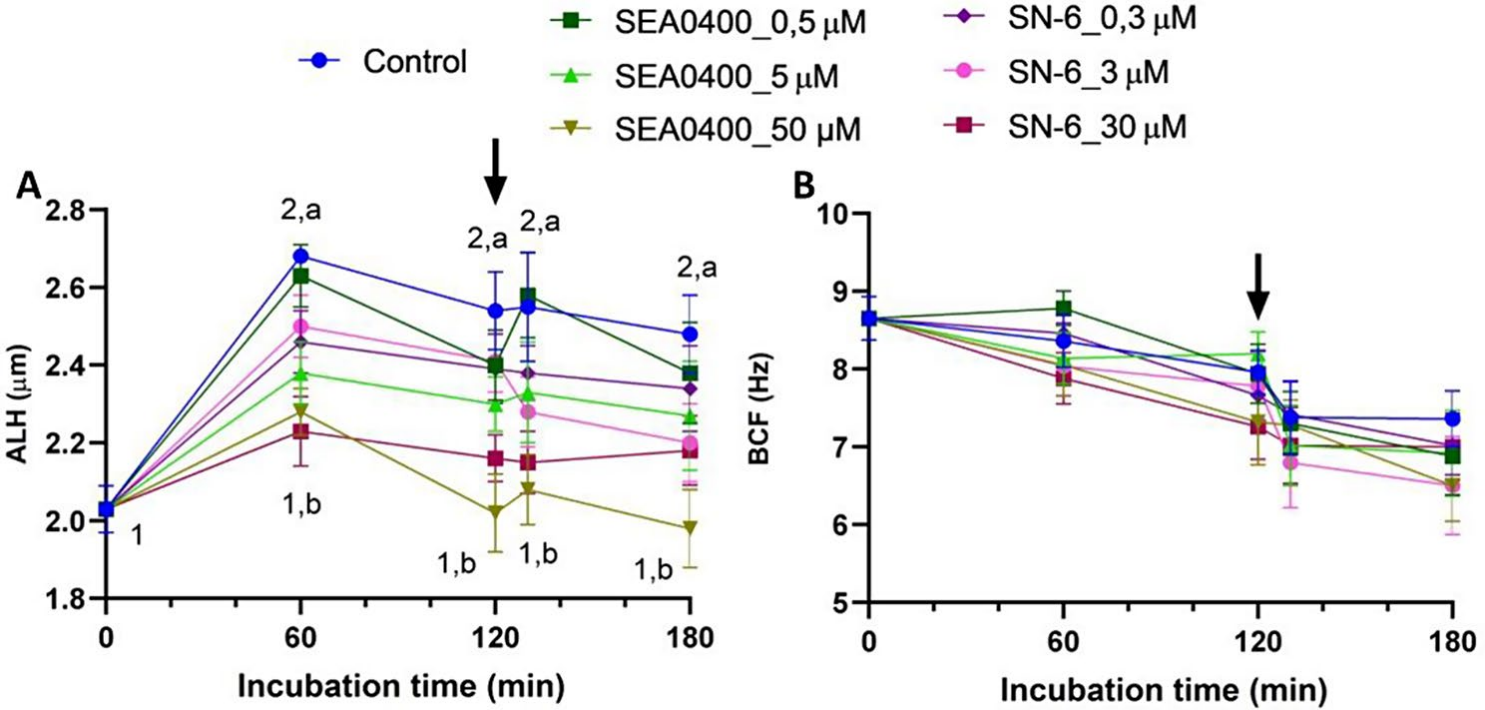
Sperm kinematics (II). Amplitude of lateral head displacement (ALH, **A**) and beat cross frequency (BCF, **B**) during in vitro capacitation of control samples and samples blocked with either SEA0400 (0.5, 5, and 50 µM) or SN-6 (0.3, 3, and 30 µM). Different superscript letters indicate significant differences between control and blocked samples within a single time point (*P* < 0.05). Different superscript numbers indicate significant differences between time points within a treatment (*P* < 0.05). The arrow indicates the addition of 10 µg/mL of progesterone at 120 min of incubation. Results are expressed as the mean ± SEM (*n* = 10)

Partial inhibition of NCX with low or intermediate concentrations of SEA0400 (0.5 or 5 µM) or SN-6 (0.3 or 3 µM) did not alter velocity parameters (VCL, VSL, and VAP). Yet, complete inhibition with 50 µM SEA0400 (*P* = 0.003) or 30 µM SN-6 (*P* = 0.017) significantly reduced these three kinematic parameters (Fig. 6). A similar effect was observed in ALH, which increased significantly during the first 60 min of incubation (*P* = 0.010); the extent of that increase was, nevertheless, smaller when NCX were completely inhibited with 50 µM SEA0400 or 30 µM SN-6 (Fig. 7). Conversely, neither BCF nor LIN, STR or WOB were found to differ between control and partially or completely NCX-blocked samples (*P* > 0.05; Suppl. Fig. 2).

### Neither partial nor complete inhibition of NCX with SEA0400 or SN-6 has an effect on membrane lipid disorder

While the percentage of viable sperm with low lipid disorder of plasma membrane decreased significantly during the first 60 min (*P* = 0.011), and that of viable sperm with high plasma membrane lipid disorder reached a transient and significant peak at 130 min (*P* = 0.008), no significant differences between control and blocked samples were observed (*P* > 0.05) (Suppl. Fig. 3).

### Blocking of NCX delays the acrosome reaction induced by progesterone, with inhibition of reverse Ca^2+^ transport having a stronger effect

**Fig. 8:**
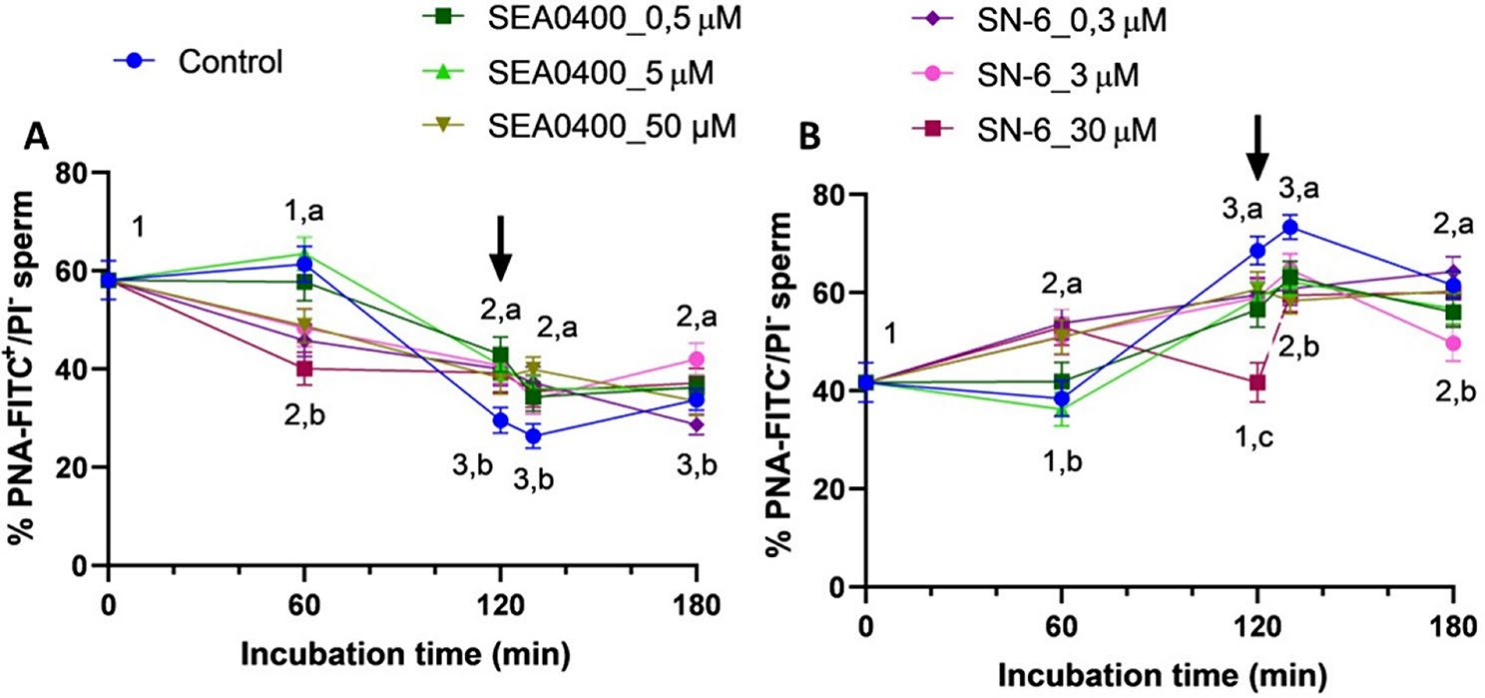
Acrosome integrity. Percentages of viable sperm with an intact acrosome (PNA-FITC+/EthD-1-, **A**) and with an exocytosed acrosome (PNA-FITC-/EthD-1-, **B**) during in vitro capacitation of control samples and samples blocked with either SEA0400 (0.5, 5, and 50 µM) or SN-6 (0.3, 3, and 30 µM). Different superscript letters indicate significant differences between control and blocked samples within a single time point (*P* < 0.05). Different superscript numbers indicate significant differences between time points within a treatment (*P* < 0.05). The arrow indicates the addition of 10 µg/mL of progesterone at 120 min of incubation. Results are expressed as the mean ± SEM (*n* = 10)

After 60 min of incubation, complete inhibition of forward Ca^2+^ transport via NCX with 50 µM SEA0400 (*P* = 0.019) and partial/complete inhibition of reverse transport with 0.3, 3 or 30 µM SN-6 (*P* = 0.002, *P* = 0.002 and *P* < 0.0001, respectively) significantly reduced the percentage of viable sperm with an intact acrosome (PNA-FITC^+^/EthD-1^−^) and increased the percentage of viable sperm with an exocytosed acrosome (PNA-FITC^+^/EthD-1^−^) (*P* = 0.005; Fig. 8). These effects were also observed when NCX were blocked with 0.5 and 5 µM SEA0400 (*P* = 0.035 and *P* = 0.009), notwithstanding they occurred later, at 120 min of incubation. Interestingly, the response of sperm to progesterone was less obvious when NCX were blocked with SEA0400 or SN-6, as the percentages of viable sperm with an exocytosed acrosome were significantly higher in the control than in the other treatments at 130 min (*P* = 0.010). These differences, nonetheless, were less clear at the end of the incubation time.

### Blocking the reverse Ca^2+^ transport via NCX with SN-6 prevents the increase in intracellular Ca^2+^ levels occurring during capacitation

**Fig. 9:**
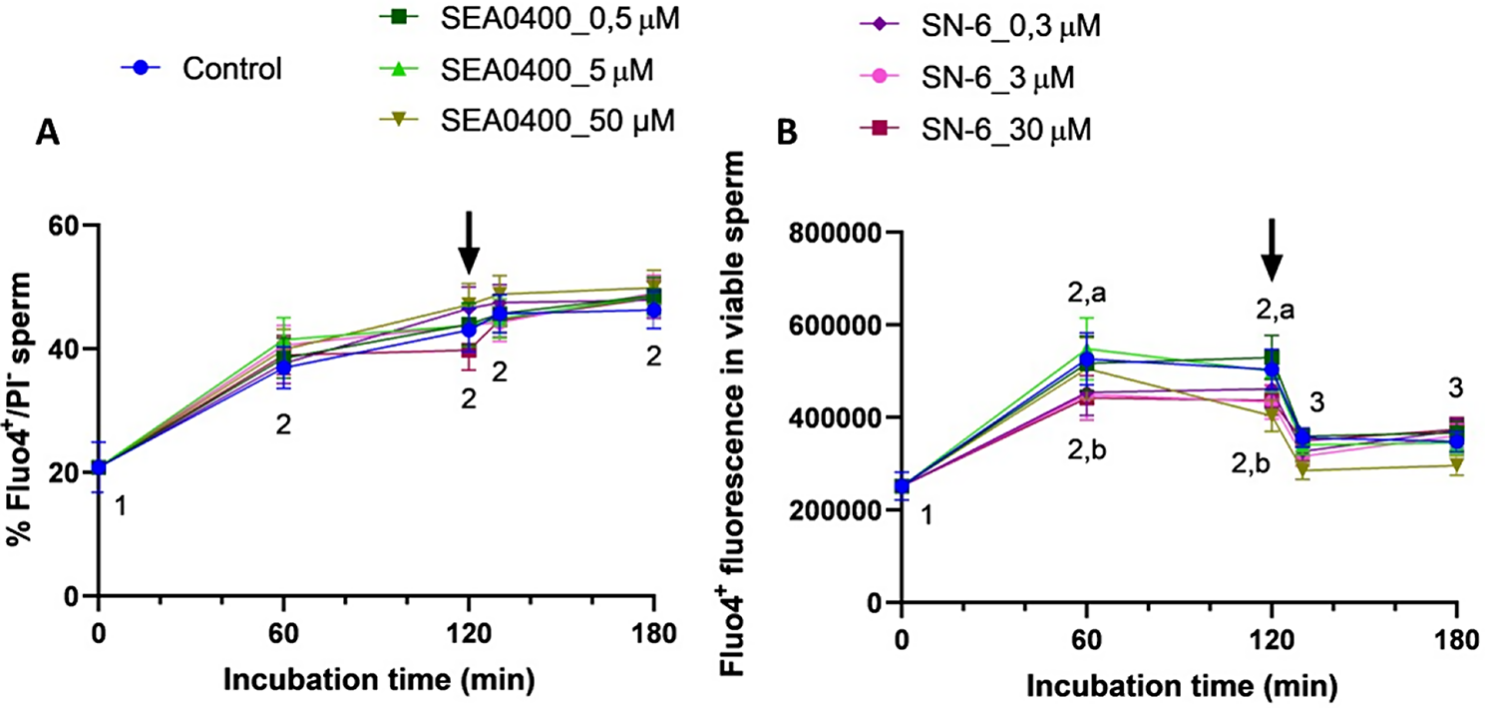
Intracellular Ca^2+^ levels. Percentages of viable sperm with high intracellular Ca^2+^ levels (Fluo4^+^/PI^−^, **A**) and fluorescence intensity of Fluo4^+^ in viable sperm (**B**) of control samples and samples blocked with either SEA0400 (0.5, 5, and 50 µM) or SN-6 (0.3, 3, and 30 µM). Different superscript letters indicate significant differences between control and blocked samples within a single time point (*P* < 0.05). Different superscript numbers indicate significant differences between time points within a treatment (*P* < 0.05). The arrow indicates the addition of 10 µg/mL of progesterone at 120 min of incubation. Results are expressed as the mean ± SEM (*n* = 10)

As expected, the percentage of viable sperm with high intracellular Ca^2+^ levels (Fluo4^+^/PI^−^) increased significantly during the first 60 min (*P* = 0.036) and maintained without significant variations (*P* > 0.05) until the end of the experiment (Fig. 9A). In contrast, at 60 min, partial/complete inhibition of reverse Ca^2+^ transport with 0.3, 3 or 30 µM SN-6 led to a significant decrease of Ca^2+^ levels (*P* = 0.023, *P* = 0.026, and *P* = 0.002, respectively) as revealed by the fluorescence intensity of Fluo4^+^ in the viable sperm population (Fluo4^+^/PI^−^; Fig. 9B). At that time point, the blockage of forward Ca^2+^ transport with SEA0400 did not affect Ca^2+^ levels (*P* > 0.05). At 120 min, intracellular Ca^2+^ levels were significantly lower in samples treated with SN-6, regardless of blocker concentration (i.e., partial and total blockage of reverse Ca^2+^ transport) (*P* = 0.012, *P* = 0.002, and *P* = 0.006, respectively), and in samples treated with 50 µM SEA0400 (i.e., complete blockage of forward Ca^2+^ transport) (*P* = 0.028) compared to the control. From 130 to 180 min incubation, the fluorescence intensity did not differ between the control and treated samples, regardless of the blocker and concentration (*P* > 0.05).

### Complete inhibition of NCX increases the percentage of sperm with low mitochondrial membrane potential (MMP)

**Fig. 10:**
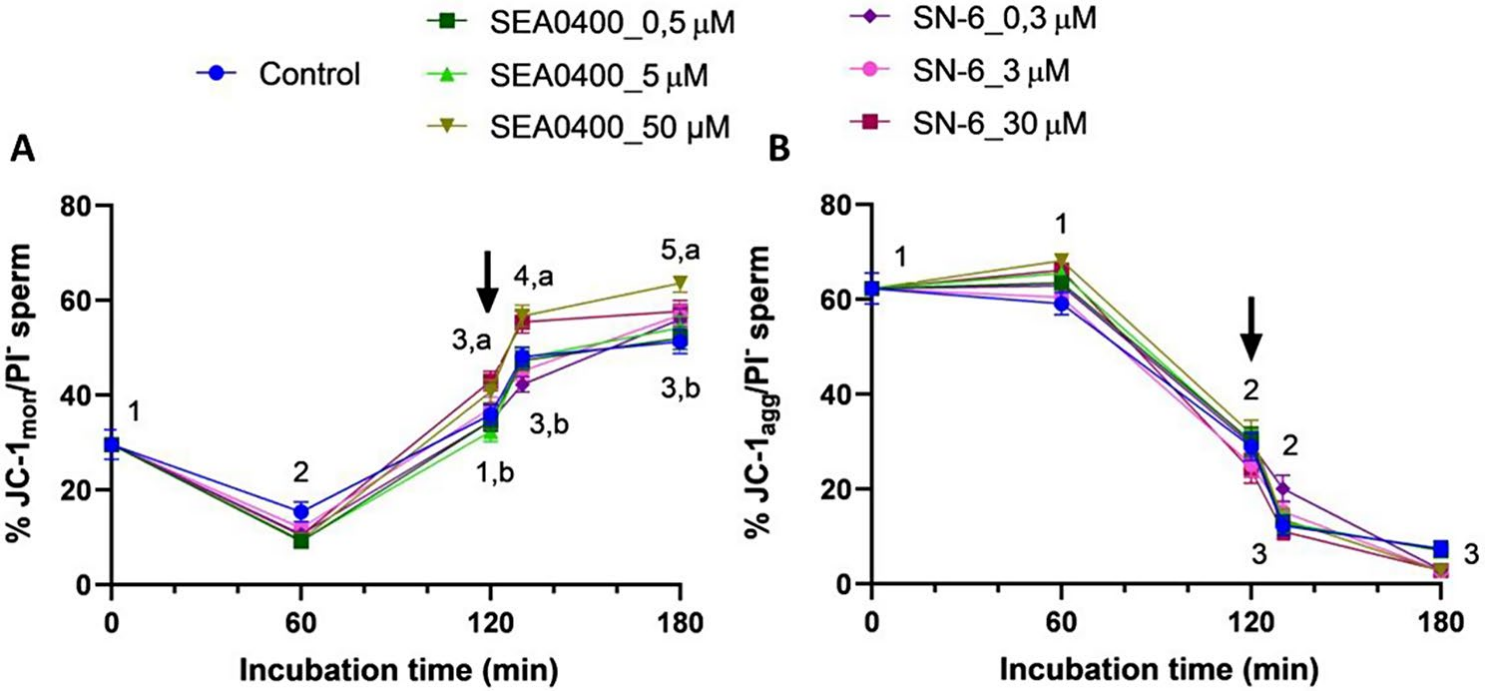
Mitochondrial membrane potential. Percentages of viable sperm with low (JC-1_mon_/PI-, **A**) and high (JC-1_agg_/PI-, **B**) mitochondrial membrane potential during in vitro capacitation of control samples and samples blocked with either SEA0400 (0.5, 5, and 50 µM) or SN-6 (0.3, 3, and 30 µM). Different superscript letters indicate significant differences between control and blocked samples within a single time point (*P* < 0.05). Different superscript numbers indicate significant differences between time points within a treatment (*P* < 0.05). The arrow indicates the addition of 10 µg/mL of progesterone at 120 min of incubation. Results are expressed as the mean ± SEM (*n* = 10)

Complete inhibition of NCX with 50 µM SEA0400 (*P* = 0.022) or 30 µM SN-6 (*P* = 0.036) significantly increased the percentage of viable sperm with low MMP (JC-1_mon_/PI^−^) after 120 min of incubation and until the end of the experiment (Fig. 10). This did not however result in a significant decrease in the percentages of viable sperm with high MMP (JC-1_agg_/PI^−^), which were similar in blocked samples and the control (*P* > 0.05).

### Partial or complete inhibition of NCX decreases total ROS levels

**Fig. 11:**
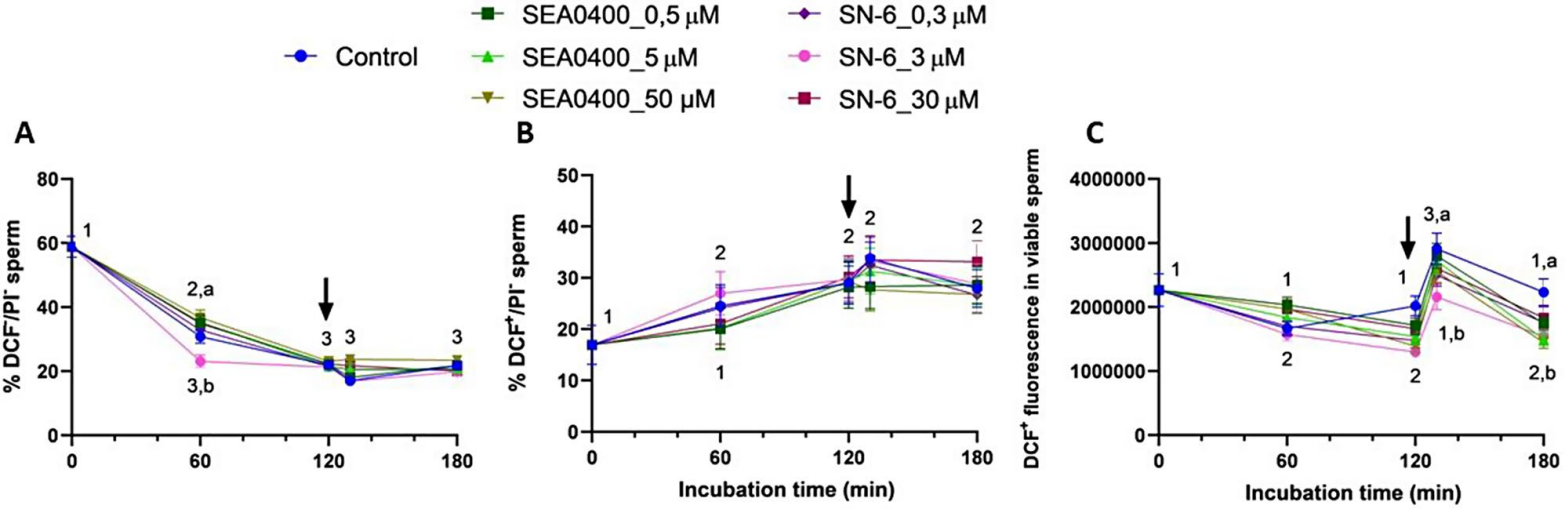
Total ROS levels. Percentages of viable sperm with low (DCF^−^/PI^−^, **A**) and high (DCF^+^/PI^−^, **B**) ROS levels and fluorescence intensity of DCF^+^ in viable sperm (**C**) during in vitro capacitation of control samples and samples blocked with either SEA0400 (0.5, 5, and 50 µM) or SN-6 (0.3, 3, and 30 µM). Different superscript letters indicate significant differences between control and blocked samples within a single time point (*P* < 0.05). Different superscript numbers indicate significant differences between time points within a treatment (*P* < 0.05). The arrow indicates the addition of 10 µg/mL of progesterone at 120 min of incubation. Results are expressed as the mean ± SEM (*n* = 10)

Although partial or complete inhibition of NCX with SEA0400 or SN-6 had no significant effect on the percentages of viable sperm with low or high ROS levels (*P* > 0.05), it did significantly decrease total ROS levels in the DCF^+^/PI^−^ sperm population (*P* = 0.013), as revealed by the fluorescence intensity of DCF^+^. In effect, the fluorescence intensity of DCF^+^ was significantly higher in the control than in blocked samples after 130 min and 180 min of incubation (*P* = 0.008 and *P* = 0.017); to note, the effects of SN-6 and SEA0400 were not concentration-dependent (Fig. 11).

### Inhibition of NCX with SN-6 or SEA0400 abolishes the transient increase of superoxide levels observed at 120 min

**Fig. 12:**
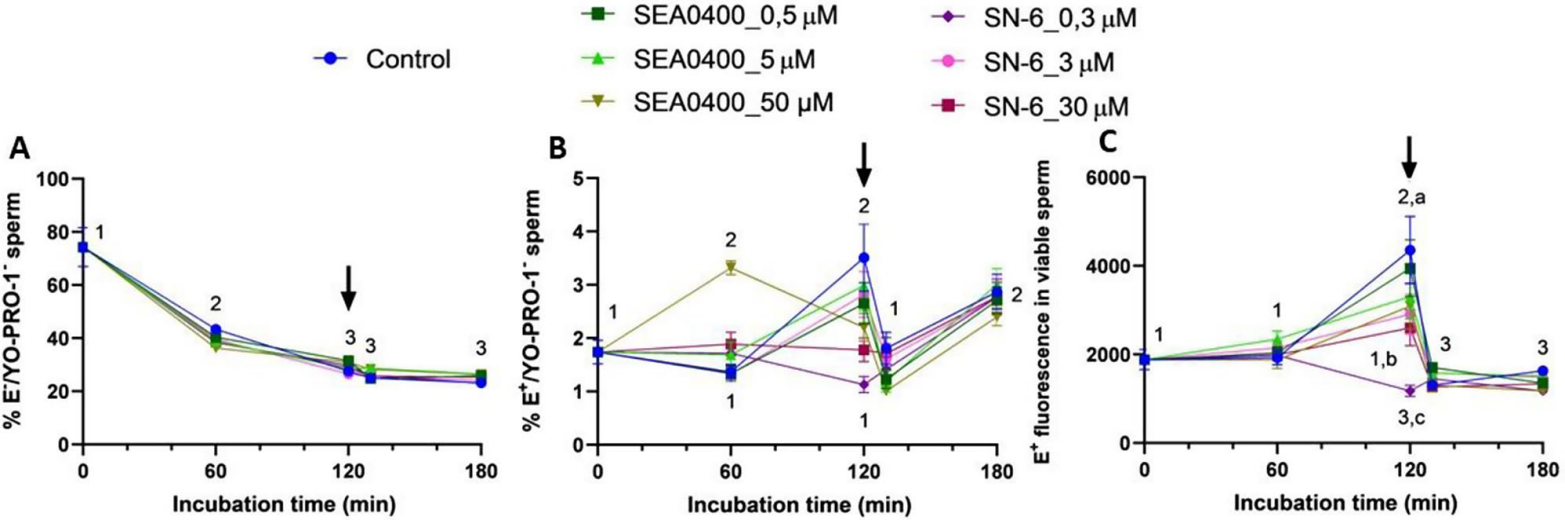
Superoxide levels. Percentages of viable sperm with low (E^−^/YO-PRO-1^−^, **A**) and high (E^+^/YO-PRO-1^−^, **B**) superoxide levels and fluorescence intensity of E^+^ in viable sperm (**C**) during in vitro capacitation of control samples and samples blocked with either SEA0400 (0.5, 5, and 50 µM) or SN-6 (0.3, 3, and 30 µM). Different superscript letters indicate significant differences between control and blocked samples within a single time point (*P* < 0.05). Different superscript numbers indicate significant differences between time points within a treatment (*P* < 0.05). The arrow indicates the addition of 10 µg/mL of progesterone at 120 min of incubation. Results are expressed as the mean ± SEM (*n* = 10)

Inhibition of forward Ca^2+^ transport with 50 µM SEA0400 significantly increased the percentage of viable sperm with high superoxide levels (E^+^/YO-PRO-1^−^) after 60 min of incubation (*P* = 0.006). At 120 min, this percentage was significantly higher in the control (*P* < 0.001) and in samples blocked with SEA0400 (*P* = 0.017) than in those incubated with SN-6 (Fig. 12). In spite of this, the percentage of viable sperm with high superoxide levels showed little differences between control and blocked samples at 130 and 180 min of incubation (*P* > 0.05). On the other hand, blocking reverse Ca^2+^ transport partially or completely with SN-6, or inhibiting the forward one with 5 or 50 µM SEA0400, abolished the increase of superoxide levels, as evaluated by the fluorescence intensity of E^+^ in viable sperm, observed at 120 min (*P* < 0.05). These differences were not seen at the end of the experiment.

### Inhibition of NCX, particularly the reverse Ca^2+^ transport, prevents the increase of tyrosine phosphorylation of sperm proteins linked to capacitation

**Fig. 13:**
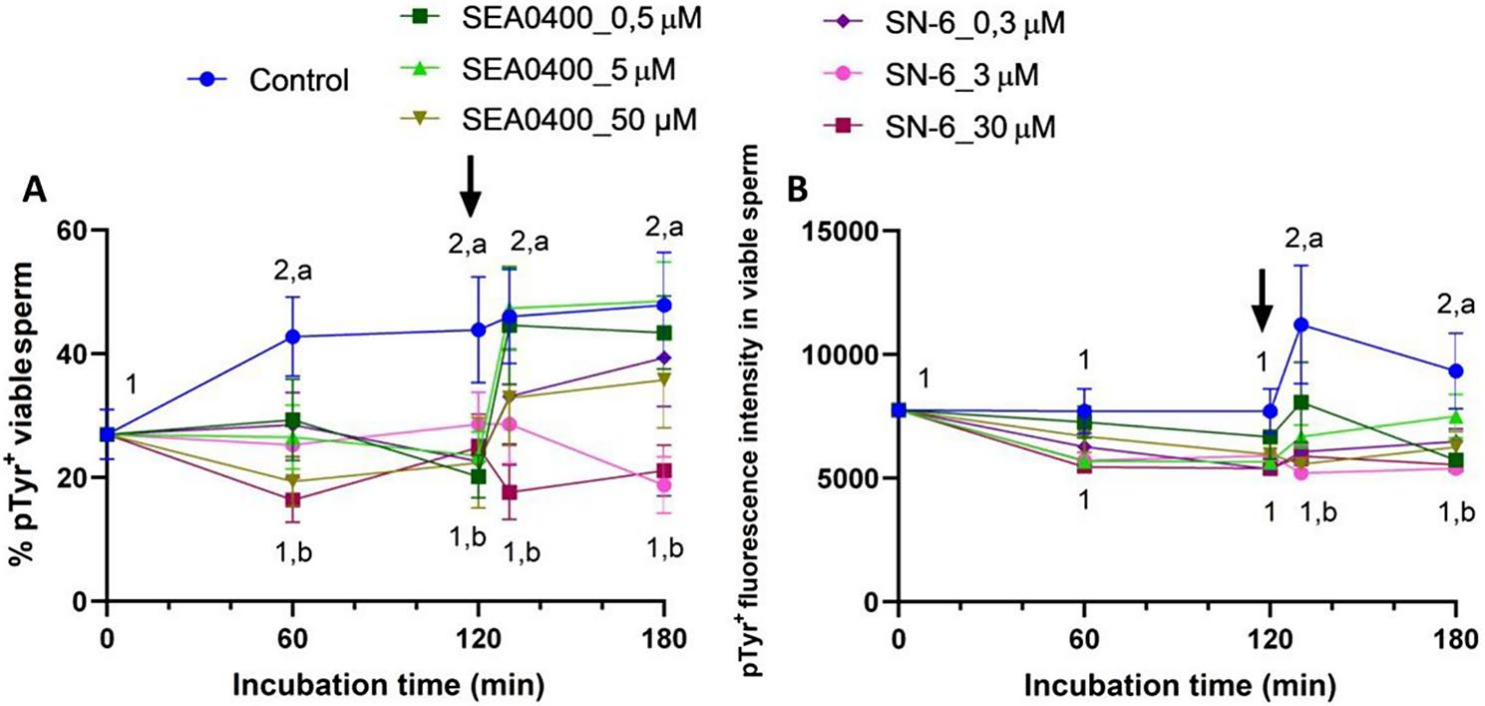
Tyrosine phosphorylation of sperm proteins. Percentages of viable sperm with phosphorylated tyrosines (pTyr^+^, **A**) and fluorescence intensity of pTyr^+^ in viable sperm (**B**) of control samples and samples blocked with either SEA0400 (0.5, 5, and 50 µM) or SN-6 (0.3, 3, and 30 µM). Different superscript letters indicate significant differences between control and blocked samples within a single time point (*P* < 0.05). Different superscript numbers indicate significant differences between time points within a treatment (*P* < 0.05). The arrow indicates the addition of 10 µg/mL of progesterone at 120 min of incubation. Results are expressed as the mean ± SEM (*n* = 10)

Inhibiting NCX significantly decreased the percentage of viable sperm with higher tyrosine phosphorylation levels (*P* < 0.001; Fig. 13A). This effect was more obvious when reverse Ca^2+^ transport was inhibited with SN-6 than when was the forward one blocked with SEA0400 (*P* = 0.005). In addition to this, the extent of that effect was greater when inhibition with the highest concentrations of the two blockers was complete (*P* < 0.001). Thus, the percentage of pTyr^+^ in viable sperm was significantly higher in the control than in blocked samples regardless of the incubation time (*P* < 0.001). In agreement with this, in the control, the fluorescence intensity of pTyr^+^ in viable sperm significantly increased after progesterone addition (130 and 180 min) (*P* < 0.001). Such an increase was not observed in samples treated with SN-6 and in those treated with the highest concentration of SEA0400 (Fig. 13B).

### SEA0400 and SN-6 block forward and reverse Ca^2+^ transport, respectively

To confirm that the differences observed in sperm under capacitation conditions resulted from the disparate blocking effect of SEA0400 and SN-6, we also analyzed the impact of these agents under non-capacitating conditions (Tris Buffer Medium [TBM] without CaCl_2_). Under such conditions, sperm variables showed little variations, and cells were unable to respond to progesterone stimulus (Suppl. Figs. 4–14). Worthy of notice is that the percentages of Fluo4^+^/PI^−^ sperm during the entire incubation period did not vary between the control (TBM) and blocked samples, even after progesterone addition (Suppl. Fig. 10). Interestingly, these percentages were significantly lower in samples incubated with 50 µM SEA0400 than in those incubated with SN-6 (*P* = 0.038). Similarly, the fluorescence intensity of Fluo4^+^ in viable sperm was significantly lower in samples blocked with SEA0400 than in those treated with SN-6 (*P* = 0.004).

## Discussion

Little data are available on the presence and localization of NCX in the plasma membrane of mammalian sperm. Our study demonstrates, for the first time, the presence of NCX1 and NCX2 in the plasma membrane of porcine sperm; immunoblotting assays showed the presence of low molecular bands corresponding to NCX3 isoforms, but immunofluorescence failed to confirm the presence of these isoforms. Immunofluorescence revealed that both NCX1 and NCX2 distributed similarly, with staining being mainly observed throughout the midpiece; nevertheless, NCX1 was observed to be more abundant than NCX2 and its distribution extended to the connecting piece. Immunoblotting analyses found that both proteins differed in their molecular weight; whereas a single band of 62 kDa was observed for NCX1, three bands of 60, 90 and 120 kDa were identified in the case of NCX2. In hamsters, only NCX1 appears to be present in the sperm plasma membrane, with three bands of 55, 70 and 110 kDa having been identified in immunoblots, whereas NCX2 and NCX3 are absent [1]. It is noticeable that the molecular weight of NCX1 shows great variability in mammals due to post-translational modifications, ranging from 60 to 120 kDa depending on the tissue and cell type [1, 37–39]. For example, while the molecular weight of NCX1 in rat hippocampus was found to be 67 kDa [39], another study in hamster brain indicated that it was of 110 kDa [1]. The molecular weight of NCX2 usually ranges between 100 kDa and 110 kDa [1, 39]; nevertheless, the presence of multiple NCX2 bands in immunoblotting assays was also reported in cardiomyocytes, with a molecular weight between 90 kDa and 115 kDa [37], and in testicular samples, where they are between 30 kDa and 50 kDa [1]. In the case of sperm, a previous study in humans demonstrated the presence of NCX in the acrosomal region and the midpiece [3]; yet, the specific NCX isoforms were not identified. In hamsters, NCX1 mainly localizes in the midpiece and, to a lesser extent, in the principal piece, and it is absent from the acrosomal region [1]. As we found that the staining in the acrosomal region was unspecific, our data would match with this previous research. In spite of this, we did not find NCX1 or NCX2 in the principal piece, as this earlier study did. These results suggest that the types and distribution of NCX isoforms may differ between mammalian species, as occurs for other ion transporters, which also exhibit species-specific differences [2, 40–42].

In the present study, we examined the role of forward and reverse transport of Ca^2+^ via NCX during sperm capacitation and acrosome reaction, through using two different pharmacological blockers, inhibiting either forward (SEA0400) or reverse transport (SN-6). The concentrations tested were chosen on the basis of the literature and after running preliminary experiments, with the aim to block these exchangers either partially or completely. According to the literature, SEA0400 inhibits forward currents through NCX partially at ≤ 5 µM and totally at ≥ 50 µM [33, 34, 43]. To note, SEA0400 has a specific inhibitory effect on NCX channels, without altering the activity of other Na^+^, Ca^2+^ and K^+^ transporters [28, 36, 44]. On the other hand, SN-6 inhibits more efficiently intracellular Ca^2+^ influx (reverse mode) than efflux (forward mode), mainly acting on NCX rather than on other ion membrane transporters [35, 36]. The blocking effect of SN-6 is also known to rely upon its concentration, with a partial NCX blockage at ≤ 10 µM [21, 36]. This different effect of SEA0400 and SN-6 on NCX activity was also observed in the present study. In our approach, the effect on intracellular Ca^2+^ levels differed between SEA0400 and SN-6. Indeed, partial blockage of forward transport (0.5 and 5 µM SEA0400) had little impact on intracellular Ca^2+^ levels. In contrast, total blockage of forward transport (50 µM SEA0400) and partial (0.3 and 3 µM SN-6) and total (30 µM SN-6) blockage of reverse transport resulted in decreased intracellular Ca^2+^ levels. These results suggest that in swine, as already reported for humans [3, 6] and rodents [4, 45], Ca^2+^ efflux during sperm capacitation does not only rely upon NCX (NCX1 and NCX2) but also on other ion transporters, potentially PMCA, notwithstanding the presence of these channels in the plasma membrane of porcine sperm has not yet been confirmed. Moreover, our study also demonstrates that both forward and reverse modes of Ca^2+^ transport via NCX are relevant for sperm capacitation in this species.

We observed that partial and total inhibition of forward and reverse transport though NCX abolished the increase in total and progressive motility after progesterone addition at 120 min, probably due to the reduced intracellular Ca^2+^ levels this time point. Moreover, an early dose-dependent effect on sperm kinematic parameters was detected during in vitro capacitation of blocked samples, especially on VCL and ALH parameters; the higher the blocker concentration the lower VCL and ALH. Altogether, these results indicate that not only complete but also partial blocking of NCX reduces the ability of sperm to hyperactivate their movement under progesterone stimulus. Remarkably, the effects of inhibiting forward and reverse transport on sperm motility were similar, thus again supporting the relevance of forward and reverse transport via NCX for Ca^2+^ homeostasis during sperm capacitation. It should be kept in mind that the transport through NCX is electrogenic (3 Na^+^:1 Ca^2+^), thus the direction of the transport does not only affect the intracellular levels of both ions but also plasma membrane potential, thus favoring either Ca^2+^ efflux or influx across the exchanger [46]. Related to this, it is worth noting that, in cardiac cells, NCX activity is also regulated by intracellular pH (pHi), the pHi increase being associated to NCX inactivation [24, 31]. In contrast, NCX regulation by direct de/phosphorylation through protein kinase A (PKA) pathway is unclear (reviewed in Morad et al. [20]). Considering that sperm capacitation is associated to an increase in the pHi and changes in membrane potential (reviewed in Delgado-Bermúdez et al. [2]), we can conclude that in porcine sperm the direction of Ca^2+^ transport via NCX differs between capacitation and acrosome reaction.

The importance of NCX in the regulation of sperm motility has been little studied, and the results seem to be inconsistent across species. In humans, blocking NCX and PMCA increases intracellular Ca^2+^ levels and reduces sperm motility and kinematics [3, 6]. This high intracellular Ca^2+^ levels reduce the ATPase activity of axonemal dynein and thus underlie the sperm motility decline [6]. In hamsters, NCX1 channels are active during sperm capacitation, thus preventing premature motility hyperactivation; in capacitated sperm, NCX1 downregulation is required for the rise in Ca^2+^ levels and further motility hyperactivation [1]. In ascidian sperm, the variations in Ca^2+^ efflux through NCX and PMCA are associated with changes in the flagellar waveform [4, 47]; whereas inhibition of NCX induces alterations in the swimming pattern [47], that of PMCA is associated to increased Ca^2+^ levels [4]. In porcine, the decline in sperm motility and kinematics observed in NCX-blocked sperm could not only result from reduced intracellular Ca^2+^ levels, but also from defective Tyr-phosphorylation of sperm proteins and impaired mitochondrial function. In agreement with this possibility, variations in Tyr-phosphorylation were seen to rely upon the concentration rather than the type of the blocker, as previously discussed for sperm motility and kinematics. Related with this, it is worth mentioning that, during sperm capacitation, the progressive increase of intracellular H^+^ and Ca^2+^ levels is associated to the activation of soluble adenylate cyclase (sAC) and protein kinase A (PKA) [48, 49]. The sAC/PKA pathway phosphorylates Tyr residues of several proteins, an essential step for membrane hyperpolarization, motility hyperactivation and acrosome reaction [50, 51].

In the present work, mitochondrial function was estimated from the analysis of mitochondrial membrane potential (MMP), and superoxide and total ROS levels. At 120 min, mitochondrial membrane potential appeared to be altered in totally blocked samples, either with SEA0400 or SN-6, but not in the partially blocked ones, whereas the impact on superoxide and total ROS levels was greater in sperm incubated with SN-6 (inhibition of reverse mode) than in those incubated with SEA0400 (inhibition of forward mode). Previous studies demonstrated that SEA0400 and SN-6 specifically block the NCX isoforms located on the plasma membrane [21]; nevertheless, the decrease of MMP after complete inhibition could indicate that at high concentrations both blockers may also affect the activity of NCLX isoforms (i.e., mitochondrial). On the other hand, in mammals, increased ROS production during sperm capacitation has been extensively reported, and it is associated to increased bicarbonate levels; however, its role in sperm capacitation and fertilization remains unclear [52, 53]. Yet, controversies exist on the ROS action during sperm capacitation; despite several studies indicating that they participate in sAC/PKA pathway activation and cholesterol efflux [52, 54, 55], recent evidence in mouse suggests that Tyr-phosphorylation through the sAC/PKA pathway is not affected by ROS levels [53]. Anomalies in sperm capacitation and fertilization are currently associated to excessive ROS production, which manifest in a decreased sperm motility [52, 54, 56, 57]. Unexpectedly, in our study, inhibition of NCX resulted in decreased rather than increased ROS production. The biological significance of these results is unclear, but we could hypothesize that this reduction in ROS levels ultimately affected the ability of capacitating sperm to fully activate the sAC/PKA pathway and phosphorylate Tyr residues of flagellar proteins.

Blocking of NCX was found to impair acrosome integrity during sperm capacitation, although this effect did not depend on the blocker type or concentration. Considering that NCX is not present in the sperm head of porcine sperm and that NCX blockage is not associated to alterations in the lipid disorder of plasma membrane, explaining these findings is difficult. A possible explanation could be that defective Tyr phosphorylation and ROS levels in NCX blocked samples could not only affect sperm motility and kinematics, but also acrosome integrity.

In conclusion, the present study confirmed the presence of NCX1 and NCX2 in the plasma membrane of porcine sperm, both exchangers differing in molecular weight, relative abundance and distribution. The physiological approach suggests that partial blockage of forward transport through NCX has little impact on the capacitation of porcine sperm, maybe due to the compensatory effect of other channels implicated in Ca^2+^ efflux. In contrast, complete inhibition of the forward transport and partial and total blockage of the reverse one through NCX affects the hyperactivation of sperm movement after progesterone stimulus, due to a defective Tyr phosphorylation of flagellar proteins and low ROS content. The decrease in intracellular Ca^2+^ levels because of the blockage of reverse transport by SN-6 suggests that Ca^2+^ entrance via NCX1 and NCX2 is relevant during sperm capacitation, at least in porcine.

## Materials and methods

All chemicals were purchased from Sigma-Aldrich (Merck KGaA, Darmstadt, Germany) unless indicated otherwise.

### Semen samples

Seminal samples (*N* = 10, each coming from a different boar) were purchased from a local farm (Gepork S.A.; Les Masies de Roda, Spain), operating under standard commercial conditions. Animals were sexually mature (between 18 and 24 months of age), from the Piétrain breed, and were lodged under standard conditions of temperature and humidity, fed a standard diet, and provided with water ad libitum. Handling of boars by the farm staff followed the guidelines for animal welfare established by the Animal Welfare Regulations issued from the Regional Government of Catalonia (Barcelona, Spain). As authors did not manipulate any animal and the seminal doses involved in the study were originally intended to artificial insemination, no specific approval from an ethics committee was needed.

Animals were collected through the gloved-hand technique. Briefly, the sperm-rich fraction of each ejaculate was immediately filtered through a gauze to remove the gel, and diluted 1:1 (v: v) in a long-term extender (Vitasem, Magapor S.L., Zaragoza, Spain) at 37 °C inside a collecting recipient. Commercial doses were obtained after further dilution and packaging into 90-mL bags at a concentration of 3 × 10^9^ sperm/dose. Seminal doses were then cooled to 17 °C, and three doses per ejaculate/animal were sent to our laboratory in a heat-insulating container at 17 °C. Once in the laboratory, sperm quality was assessed to ensure that all seminal doses fulfilled the minimum quality standards (viable sperm ≥ 80%; total motile sperm ≥ 70%; and morphologically normal sperm ≥ 85% [42]).

### Experimental design

The presence of NCX1, NCX2 and NCX3 in the sperm plasma membrane was determined through immunofluorescence and immunoblotting. Following this, the physiological role of NCX during in vitro capacitation and acrosome reaction was analyzed by blocking the forward and reverse transport of Ca^2+^ via these exchangers with 2-[4-[(2,5-difluorophenyl)methoxy]phenoxy]-5-ethoxyaniline (SEA0400) and ethyl 2-[[4-[(4-nitrophenyl)methoxy]phenyl]methyl]-1,3-thiazolidine-4-carboxylate (SN-6), respectively. Pharmacological blockers were added at the beginning of the experiment (0 min). Three concentrations of each blocking agent, aiming at achieving partial and complete inhibition, were tested. These concentrations, which were 0.5, 5 and 50 µM for SEA0400, and 0.3, 3 and 30 µM for SN-6, were established on the basis of preliminary experiments and the literature [28, 35, 36, 44].

For each independent experiment, the three seminal doses coming from the same ejaculate/animal were pooled and centrifuged at 600× g and 17 °C for 5 min; sperm pellets were then resuspended in capacitating medium (TCM: 20 mM HEPES, 100 mM NaCl, 3.1 mM KCl, 5 mM glucose, 21.7 mM sodium L-lactate, 1 mM sodium pyruvate, 0.3 mM Na_2_HPO_4_, 0.4 mM MgSO_4_·7 H_2_O, 4.5 mM CaCl_2_·2 H_2_O, 5 mg/mL bovine serum albumin (BSA), and 15 mM sodium bicarbonate) to a final concentration of 1 × 10^7^ sperm/mL. Aliquots were distributed into control samples (without blocker) and blocked samples (with either SEA0400 or SN-6 at the aforementioned concentrations). Samples were incubated at 38.5 °C, 100% humidity and 5% CO_2_ (Binder GmbH, Tuttlingen, Germany) for 180 min; in all samples, 10 µg/mL progesterone was added at 120 min. Analysis of sperm variables was conducted after 0, 60, 120, 130, and 180 min of incubation.

At each relevant time point, sperm motility and kinematics were assessed with a computer-assisted sperm analysis (CASA) system, whereas membrane lipid disorder, acrosome integrity, mitochondrial membrane potential, tyrosine phosphorylation of sperm proteins, and intracellular levels of Ca^2+^, reactive oxygen species (ROS) and superoxides were determined by flow cytometry.

To confirm that SEA0400 blocks the forward transport and SN-6 blocks the reverse transport of NCX channels, the same set of experiments was conducted in sperm samples incubated under non-capacitating conditions (Tris Buffer Medium, TBM: 20 mM HEPES, 112 100 mM NaCl, 4.7 mM KCl, 5 mM glucose, 21.7 mM sodium L-lactate, 1 mM sodium pyruvate, 0.3 mM Na_2_HPO_4_, and 0.4 mM MgSO_4_·7 H_2_O). Each individual experiment included a positive control (sperm incubated in TCM without any blocker), a negative control (sperm incubated in TBM without any blocker) and blocked samples (sperm incubated in TBM with either 50 µM SEA0400 or 0.3, 3 or 30 µM SN-6). Sperm variables were analyzed at 0, 60, 120, 130, and 180 min of incubation, as previously indicated. Results are shown in Supplementary Figs. 4–14.

### Immunoblotting

Immunoblotting assays were conducted following a previously described protocol [58]. Briefly, for total protein extraction, sperm pellets were resuspended in RIPA lysis buffer (R0278), supplemented 1:100 (v: v) with a commercial protease inhibitor cocktail (P8340) containing 0.1 mM phenyl-methane-sulfonylfluoride (PMSF) and 700 mM sodium orthovanadate. Samples were then incubated in agitation at 4 °C for 30 min, sonicated on ice three times (five pulses each; 20 kHz) every 2 min, and centrifuged at 10,000× g and 4 °C for 15 min to collect supernatants. Quantification of total protein in supernatants was carried out in triplicate using a detergent compatible (DC) method (BioRad, Hercules, CA, USA). Once quantified, samples were diluted to 1 µg total protein/µL in lysis buffer; 10 µL of each sample were mixed with 10 µL of 4× Laemmli reducing buffer containing 5% β-mercaptoethanol (BioRad). Samples were incubated at 95 °C for 5 min and loaded onto 12% polyacrylamide gels (Mini-PROTEAN^®^ TGX™ Precast Gels, BioRad). Gels were run at 20 mA and 120–150 V through an electrophoretic system (IEF Cell Protean System, BioRad). Proteins from gels were then transferred onto polyvinylidene fluoride membranes using a Trans-Blot^®^ Turbo™ device (BioRad). Thereafter, protein bands were visualized under UV exposition and scanned using a G: BOX Chemi XL system (SynGene, Frederick, MD, USA). Membranes were blocked with 1× TBS containing 10 mM Tris (Panreac, Barcelona, Spain), 150 mM NaCl (LabKem, Barcelona, Spain), 0.05% (w: v) Tween-20 (pH adjusted to 7.3; Panreac, Barcelona, Spain), and 5% bovine serum albumin (BSA, Roche Diagnostics, S.L.; Basel, Switzerland) at room temperature and agitation for 1 h.

Membranes were subsequently incubated with specific primary antibodies against NCX1 (SLC8A1), NCX2 (SLC8A2), or NCX3 (SLC8A3) (Alomone Labs, Jerusalem, Israel), which were previously diluted in blocking solution at 1:2,000 (v: v), at 4 °C overnight under agitation. Membranes were then rinsed three times with washing solution (1× TBS-Tween20), and incubated at room temperature under agitation for 1 h with an anti-rabbit secondary antibody conjugated with horseradish peroxidase (ref. P0448, Agilent, Santa Clara, CA, USA) diluted at 1:5,000 (v: v) in blocking solution. Membranes were rinsed five times with washing solution and protein bands were visualized with a chemiluminescent substrate (Immobilion™ Western Detection Reagents; Millipore, Darmstadt, Germany) and scanned with G: BOX Chemi XL 1.4 (SynGene, Cambridge, UK). The specificity of primary antibodies was confirmed through peptide competition assays using a specific blocking peptide for each primary antibody (Alomone Labs) at a concentration five times higher than the primary antibody.

### Immunofluorescence

Sperm samples were washed with PBS (pH = 7.3) at 500× g and room temperature for 5 min, and then fixed with 4% (w: v) paraformaldehyde at room temperature for 30 min. After fixation, samples were washed twice with PBS at 500× g and room temperature for 5 min, and resuspended in PBS (final concentration: 5 × 10^6^ sperm/mL). Next, 150 µL of each sperm sample was placed onto ethanol-rinsed slides and incubated at room temperature for 1 h to promote cell adhesion. Adhered sperm cells were permeabilized by incubation with 1% Triton X-100 in PBS. Next, antigens were unmasked according to the protocol of Kashir et al. [59]. In brief, slides were exposed to acidic Tyrode’s solution for 20 s, and the acid was then neutralized by washing three times with neutralization solution (Tris 100 mM, pH = 8.5), and three times with PBS.

To block nonspecific binding sites, samples were incubated with a blocking solution consisting of 5% BSA in PBS at room temperature for 1 h. Subsequently, sperm were incubated with primary NCX antibodies, either NCX1, NCX2 or NCX3 (Alomone Labs), diluted 1:100 (v: v) at room temperature for 1 h. After washing five times with PBS (5 min per wash), samples were incubated with a secondary anti-rabbit antibody Alexa Fluor™ Plus 488 (ref. A32731, Invitrogen, Waltham, MA, USA) diluted 1:200 (v: v) in blocking solution at room temperature for 1 h. Samples were again washed five times with PBS (5 min per wash), air dried and mounted with 10 µL of ProLongTM Glass Antifade Mountant with NucBlue™ (Hoechst 33342; ref. P36985, Invitrogen) in the dark. Specificities of primary antibodies were confirmed through peptide competition assays using a specific blocking peptide for each primary antibody; in all cases, the blocking peptide was five times in excess with regard to the primary antibody.

Sperm were examined under a confocal microscope (CLSM Nikon A1R; Nikon Corp, Tokyo, Japan). Samples were excited at 405 nm to localize the Hoechst 33,342-stained nuclei, and then at 488 nm to determine the localization of NCX1, NCX2, and NCX3.

### Evaluation of sperm motility

Sperm motility was evaluated using a CASA system, which consisted of a phase contrast microscope (Olympus BX41; Olympus, Tokyo, Japan) equipped with a warmed stage, a video camera and the ISAS software (Integrated Sperm Analysis System V1.0; Proiser SL, Valencia, Spain). Three µL of each sample was placed into a prewarmed (38 °C) Leja chamber (IMV Technologies, L’Aigle, France) and observed under a negative phase-contrast field (Olympus 10 × 0.30 PLAN objective). At least 1,000 sperm were examined per replicate, and three replicates per sample were evaluated.

For each sperm sample and concentration of inhibitor, percentages of total and progressively motile sperm were determined. Furthermore, different sperm kinematic parameters, including curvilinear velocity (VCL, µm/s); straight line velocity (VSL, µm/s); average path velocity (VAP, µm/s); amplitude of lateral of head displacement (ALH, µm); beat cross frequency (BCF, Hz); linearity (LIN, %), which was calculated assuming that LIN = VSL/VCL × 100; straightness (STR, %), resulting from VSL/VAP × 100; and motility parameter wobble (WOB, %), obtained from VAP/VCL × 100, were measured. A sperm cell was classified as motile when its VAP was equal to or greater than 10 μm/s and progressively motile when its STR was equal to or greater than 45%. For each treatment and incubation time, motility parameters were expressed as the mean ± standard error of the mean (SEM; *n* = 10).

### Flow cytometry

Flow cytometry was used to determine membrane lipid disorder, acrosome integrity, mitochondrial membrane potential (MMP), tyrosine phosphorylation of sperm proteins, and intracellular levels of Ca^2+^, reactive oxygen species (ROS) and superoxides. Each sperm parameter was evaluated with a proper combination of fluorochromes, all being purchased from ThermoFisher Scientific (Waltham, MA, USA). Before staining, all samples were diluted to a final concentration of 1 × 10^6^ sperm/mL and incubated at 38 °C in the dark after the addition of the corresponding fluorochromes. For each parameter, a total of three replicates per sample were examined.

Samples were evaluated using a CytoFLEX cytometer (Beckman Coulter; Fullerton, CA, USA). All samples were excited with a blue laser (488 nm). The FITC filter (525/40) was used for YO-PRO-1, PNA-FITC, Fluo4, JC-1 monomers (JC-1_mon_) and 2’,7’-dichlorofluorescein (DCF) fluorochromes. The PE filter (585/42) was utilized to detect ethidium (E), JC-1 aggregates (JC-1_agg_) and merocyanine 540 (M540) fluorochromes. APC (660/20) and PC5.5 (690/50) filters were employed for AlexaFluor647-conjugated anti-pTyr antibody and propidium iodide (PI), respectively. Flow rate and gain were not altered throughout the experiment.

#### Membrane lipid disorder

Lipid disorder of sperm plasma membrane was evaluated following the protocol of Rathi et al. [60], as modified by Yeste et al. [61]. Sperm were incubated with M540 (10 nM) and YO-PRO-1 (31.25 nM) at 38 °C for 10 min. M540 is a hydrophobic fluorochrome that can intercalate within the membrane. As membrane fluidity increases M540 uptake, this fluorochrome is considered as a reliable marker for destabilization of sperm plasma membrane, and has been validated in many species, including the porcine [62]. YO-PRO-1 is a vital stain that only labels sperm with an increased membrane permeability. Four sperm populations were identified: (1) viable sperm with low membrane lipid disorder (M540^−^/YO-PRO-1^−^), (2) viable sperm with high membrane lipid disorder (M540^+^/YO-PRO-1^−^), (3) non-viable sperm with low membrane lipid disorder (M540^−^/YO-PRO-1^+^), and (4) non-viable sperm with high membrane lipid disorder (M540^+^/YO-PRO-1^+^). Results are expressed as the percentage viable sperm with low (M540^−^/YO-PRO-1^−^) and high (M540^+^/YO-PRO-1^−^) membrane lipid disorder (mean ± SEM; *n* = 10).

#### Acrosome integrity

Acrosome integrity was evaluated following the modified protocol of Cooper and Yeung [63]. Samples were incubated with LIVE/DEAD working solution (Thermo Fisher Scientific, Massachusetts, USA) at 38 °C for 20 min in the dark, then centrifuged at 1,000× g and room temperature for 3 min. Samples were subsequently resuspended in blocking solution (PBS + 4 mg/mL bovine serum albumin, BSA) and centrifuged again at 1,000× g for 3 min. Pellets were resuspended in ice-cold methanol for 30 s, centrifuged at 1,000× g for 3 min, and resuspended again in 250 µL PBS; PNA-FITC (final concentration: 1.17 µM) was immediately added to resuspended samples, which were then incubated at 38 °C in the dark for 15 min. After incubation, samples were centrifuged at 1,000× g for 3 min and pellets were resuspended in 150 µL PBS. Four sperm populations were identified in dot-plots: (1) viable sperm with an intact acrosome (PNA-FITC^+^/PI^−^), (2) viable sperm with an exocytosed acrosome (PNA-FITC^−^/PI^−^), (3) non-viable sperm with an intact acrosome (PNA-FITC^+^/PI^+^), and (4) non-viable sperm with an exocytosed acrosome (PNA-FITC^−^/PI^+^). Results are expressed as the percentage of viable sperm (PI^−^) with either an intact (PNA-FITC^+^) or an exocytosed acrosome (PNA-FITC^−^) (mean ± SEM; *n* = 10).

#### Mitochondrial membrane potential (MMP)

Determination of mitochondrial membrane potential (MMP) was performed through staining with JC-1 (final concentration: 750 nM), diluted at 1:8,000 (v: v) in PBS, and fixable far-red LIVE/DEAD [64]. After staining, samples were incubated at 38 °C in the dark for 30 min. High MMP results in JC-1 aggregation (JC-1_agg_) and the subsequent emission of red fluorescence; in contrast, in sperm cells with low MMP, JC-1 remains as a monomer (JC-1_mon_) and emits green fluorescence (JC-1^−^). Four populations were distinguished: (1) viable sperm with low MMP (JC1_mon_/PI^−^), (2) viable sperm with high MMP (JC-1_agg_/PI^−^), (3) non-viable sperm with low MMP (JC-1_mon_/PI^+^), and (4) non-viable sperm with high MMP (JC1_agg_/PI^+^). For each treatment and incubation time, results are expressed as percentages of viable sperm with low (JC-1_mon_/PI^−^) and high (JC-1_agg_/PI^−^) MMP (mean ± SEM; *n* = 10).

#### Intracellular levels of Ca^2+^

Intracellular levels of Ca^2+^ were evaluated through double staining with Fluo4-AM and PI. Fluo4-AM is able to penetrate sperm cells, bind Ca^2+^ and emit green fluorescence. Sperm were incubated with Fluo4-AM (final concentration: 1.17 µM) and PI (final concentration: 5.6 µM) at 38 °C for 10 min. Four sperm populations were identified in the dot-plots: (1) viable sperm with low Ca^2+^ levels (Fluo4^−^/PI^−^), (2) viable sperm with high Ca^2+^ levels (Fluo4^+^/PI^−^), (3) non-viable sperm with low Ca^2+^ levels (Fluo4^−^/PI^+^), and (4) non-viable sperm with high Ca^2+^ levels (Fluo4^+^/PI^+^). Data are shown as percentages of viable sperm with high Ca^2+^ levels (Fluo4^+^/PI^−^), and the geometric mean intensity of Fluo4 in the Fluo4^+^/PI^−^ population (mean ± SEM; *n* = 10).

#### Intracellular levels of reactive oxygen species (ROS)

Intracellular levels of total ROS were determined through staining with 2’,7’-dichlorodihydrofluorescein diacetate (H_2_DCFDA) and PI, following the protocol of Guthrie and Welch [65] with minor modifications. Briefly, sperm were incubated with H_2_DCFDA (final concentration: 350 nM) at 38 °C for 20 min in the dark, and then with PI (final concentration: 6 µM) at the same conditions for further 5 min. H_2_DCFDA is a non-fluorescent agent that can enter the sperm cell and react with ROS, thus converting into 2’,7’-dichlorofluorescein (DCF^+^), a green-fluorescent molecule. The following four populations were distinguished in dot plots: (1) viable sperm with low ROS levels (DCF^−^/PI^−^), (2) viable sperm with high ROS levels (DCF^+^/PI^−^), (3) non-viable sperm with low ROS levels (DCF^−^/PI^+^), and (4) non-viable sperm with high ROS levels (DCF^+^/PI^+^). Results are expressed as percentages of viable sperm with low (DCF^−^/PI^−^) and high ROS levels (DCF^+^/PI^−^), and the geometric mean of DCF^+^-fluorescence intensity in the DCF^+^/PI^−^ population (mean ± SEM; *n* = 10).

#### Intracellular levels of superoxides

Intracellular levels of superoxides (O_2_^•−^) were evaluated through double-staining with hydroethidine (HE) and YO-PRO-1 [65]. Sperm were incubated with 5 µM HE and 25 nM YO-PRO-1 at 38 °C in the dark for 20 min. HE permeates the sperm plasma membrane and is oxidized into ethidium (E^+^), which emits red fluorescence, by O_2_^•−^. Again, four separate populations were identified in dot-plots: (1) viable sperm with low superoxide levels (E^−^/YO-PRO-1^−^), (2) viable sperm with high superoxide levels (E^+^/YO-PRO-1^−^), (3) non-viable sperm with low superoxide levels (E^−^/YO-PRO-1^+^), and (4) non-viable sperm with high superoxide levels (E^+^/YO-PRO-1^+^). Results are expressed as percentages of viable sperm with low (E^−^/YO-PRO-1^−^) and high superoxide levels (E^+^/YO-PRO-1^−^), and the geometric mean of E^+^-fluorescence intensity in the E^+^/YO-PRO-1^−^ population (mean ± SEM; *n* = 10).

#### Tyrosine-phosphorylation of sperm proteins

Analysis of tyrosine-phosphorylation (pTyr) of sperm proteins was conducted as described in Peris-Frau et al. [66]. Three separate tubes were prepared. Sperm were first stained with the far-red LIVE/DEAD fluorochrome (ThermoFisher Scientific) at 38 °C in the dark for 20 min. Samples were subsequently centrifuged at 1,000× g and room temperature for 3 min, resuspended in 10 mL blocking buffer (5% BSA in PBS), incubated for 1 min and centrifuged again at the same conditions. Then, sperm pellets were resuspended in 4% paraformaldehyde and incubated at room temperature for 15 min. Samples were then centrifuged at 1,000× g and room temperature for 3 min, resuspended in PBS and stored at 4 °C overnight. Thereafter, samples were centrifuged at 1,000× g and room temperature for 3 min, and resuspended in permeabilization buffer (0.5 g BSA, 100 µL Triton X-100, and 0.02 g sodium azide in 10 mL PBS) and incubated at room temperature for 60 min. After centrifugation at 1,000× g and room temperature for 3 min, sperm pellets from two of the three tubes were resuspended with the antibody solution (blocking solution with anti-pTyr antibody conjugated with AlexaFluor647 (Abcam) at 1:1,000). The third tube was incubated with blocking solution in the absence of the antibody, and all tubes were incubated together, at 4 °C overnight and agitation in the dark. Samples were centrifuged at 1,000× g and room temperature for 3 min, resuspended in PBS and analyzed with the flow cytometer. Four populations were identified: (1) viable sperm with low pTyr levels, (2) viable sperm with high pTyr levels, (3) non-viable sperm with low pTyr levels, and (4) non-viable sperm with high pTyr levels. Results are expressed as percentages of viable sperm with high pTyr levels (pTyr^+^/viable sperm), and fluorescence intensity of pTyr in the pTyr^+^/viable sperm population (mean ± SEM; *n* = 10).

#### Statistical analyses

Data were analyzed using a statistical package (IBM SPSS Statistics 27.0; Armonk, New York, NY, USA). Normal distribution of data and homogeneity of variances were checked with Shapiro-Wilk and Levene tests, respectively. Following this, a linear mixed model was run with each sperm parameter being considered the independent variable. The intrasubject factor was the incubation time (0, 60, 120, 130, and 180 min), and the intersubject factor was the treatment (control and samples blocked with either SEA0400 or SN-6 at the aforementioned concentrations). The post-hoc Sidak test was used for pairwise comparisons. The level of significance was set at *P* ≤ 0.05 in all analyses, and data are shown as mean ± SEM.

### Electronic supplementary material

Below is the link to the electronic supplementary material.

Supplementary Material 1

## Data Availability

Raw data supporting the findings of this study are available from the corresponding author on request.
