# Supplementary material for: Inhibition of forward and reverse transport of Ca2+ via Na+/Ca2+ exchangers (NCX) prevents sperm capacitation
Source: Biol Res. 2024 Aug 23;57:57. doi: 10.1186/s40659-024-00535-9 (PMC11342557; doi:10.1186/s40659-024-00535-9)
Supplement: Supplementary file 1 — Supplementary Material 1 [file 40659_2024_535_MOESM1_ESM.docx]

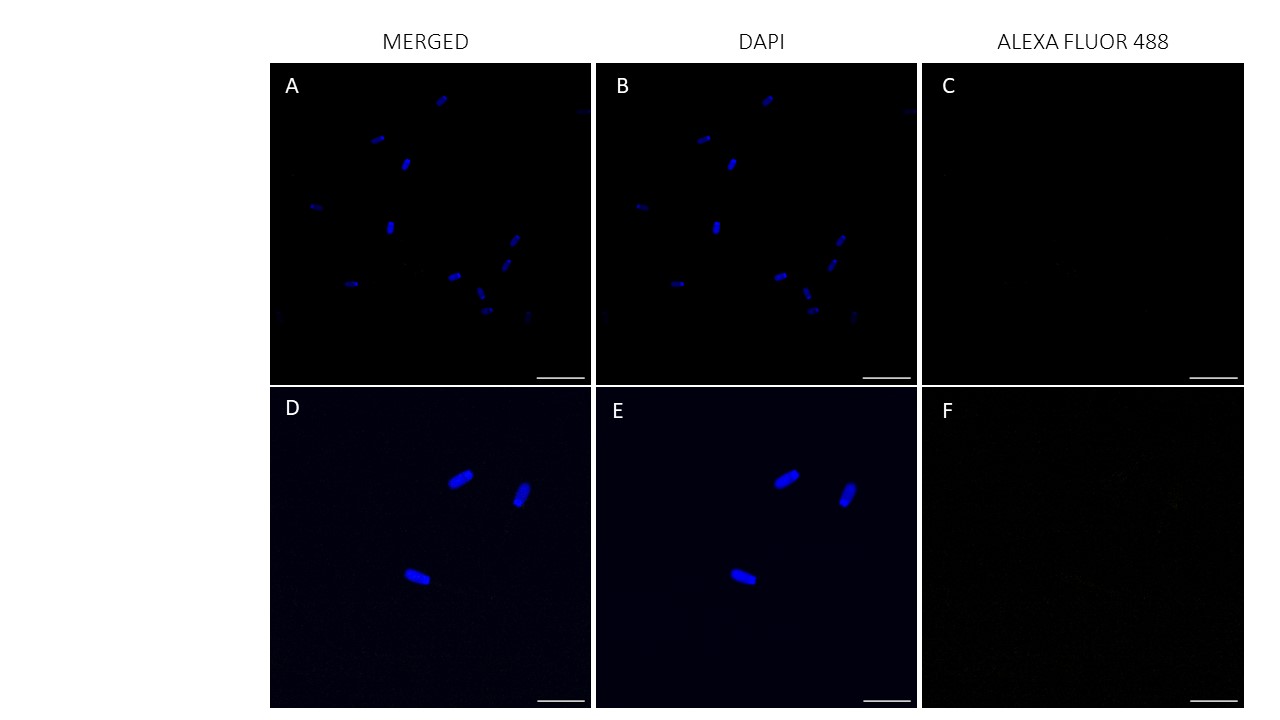


Suppl. Fig. 1. Negative controls for immunolocalization assays against NCX channels. Nuclei appear blue-stained by DAPI (4′6′-diamidion-2-phenylindole). Scale bar: 30 µm (A-C) and 15 µm (D-F).


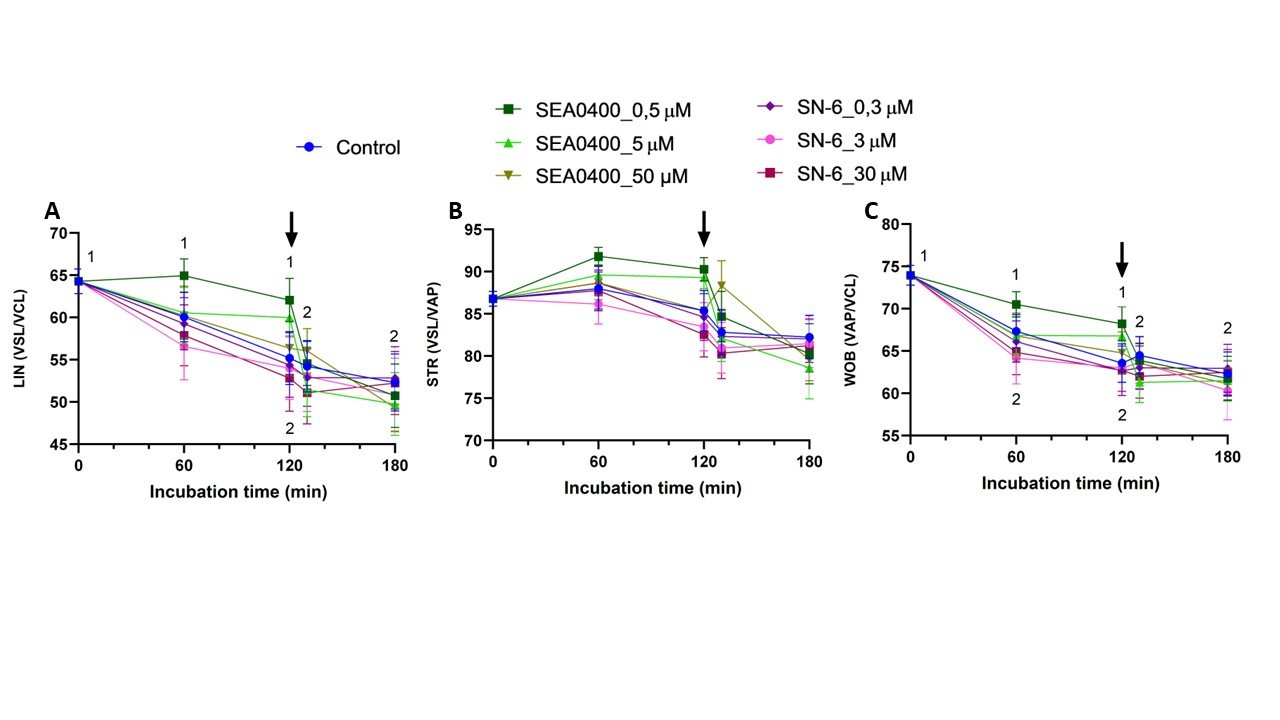


Suppl. Fig. 2. LIN (A), STR (B), and WOB (C) during in vitro capacitation of control samples and samples blocked with either SEA0400 (0.5, 5, and 50 μM) or SN-6 (0.3, 3, and 30 μM). Different superscript letters indicate significant differences between control and blocked samples within a single time point (*P* < 0.05). Different superscript numbers indicate significant differences between time points within a treatment (*P* < 0.05). The arrow indicates the addition of 10 μg/mL of progesterone at 120 min of incubation. Results are expressed as the mean ± SEM.


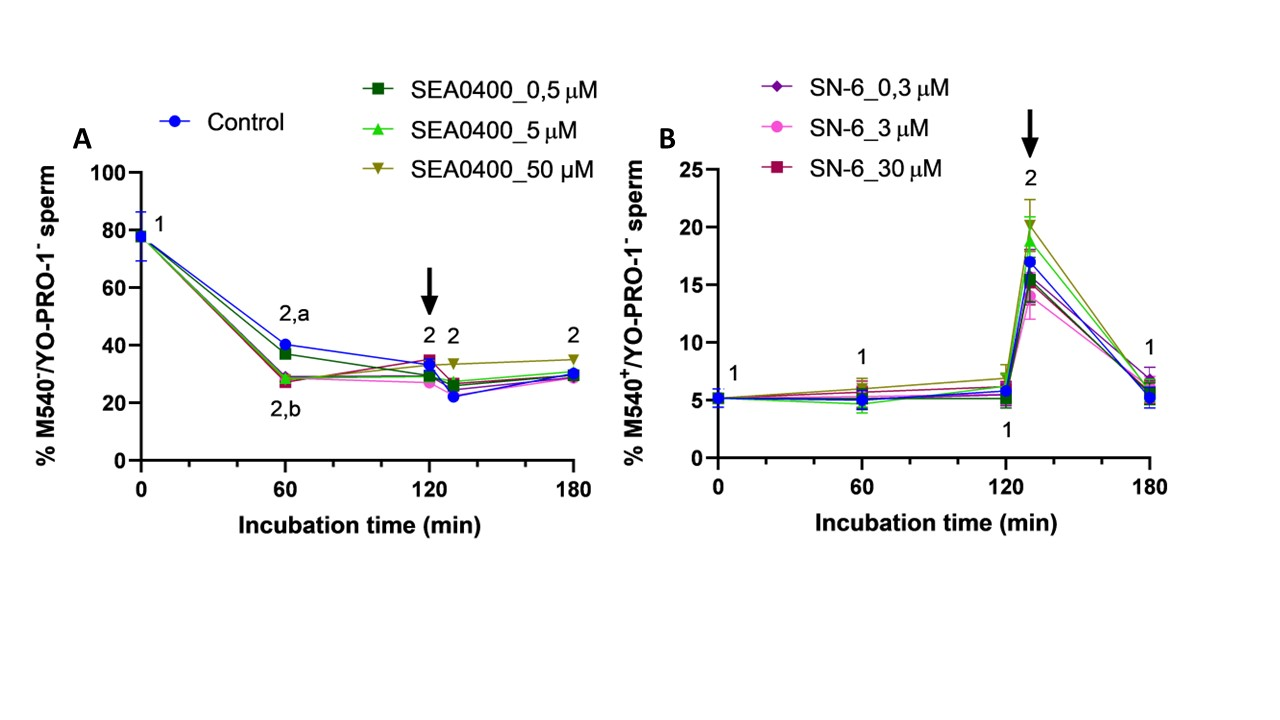


Suppl. Fig. 3**.** Lipid disorder of plasma membrane. Percentages of viable sperm with low (M540^-^/YO-PRO-1^-^, A) and high (M540^+^/YO-PRO-1^-^, B) lipid disorder of plasma membrane during in vitro capacitation of control samples and samples blocked with either SEA0400 (0.5, 5, and 50 μM) or SN-6 (0.3, 3, and 30 μM). Different superscript letters indicate significant differences between control and blocked samples within a single time point (*P* < 0.05). Different superscript numbers indicate significant differences between time points within a treatment (*P* < 0.05). The arrow indicates the addition of 10 μg/mL of progesterone at 120 min of incubation. Results are expressed as the mean ± SEM.


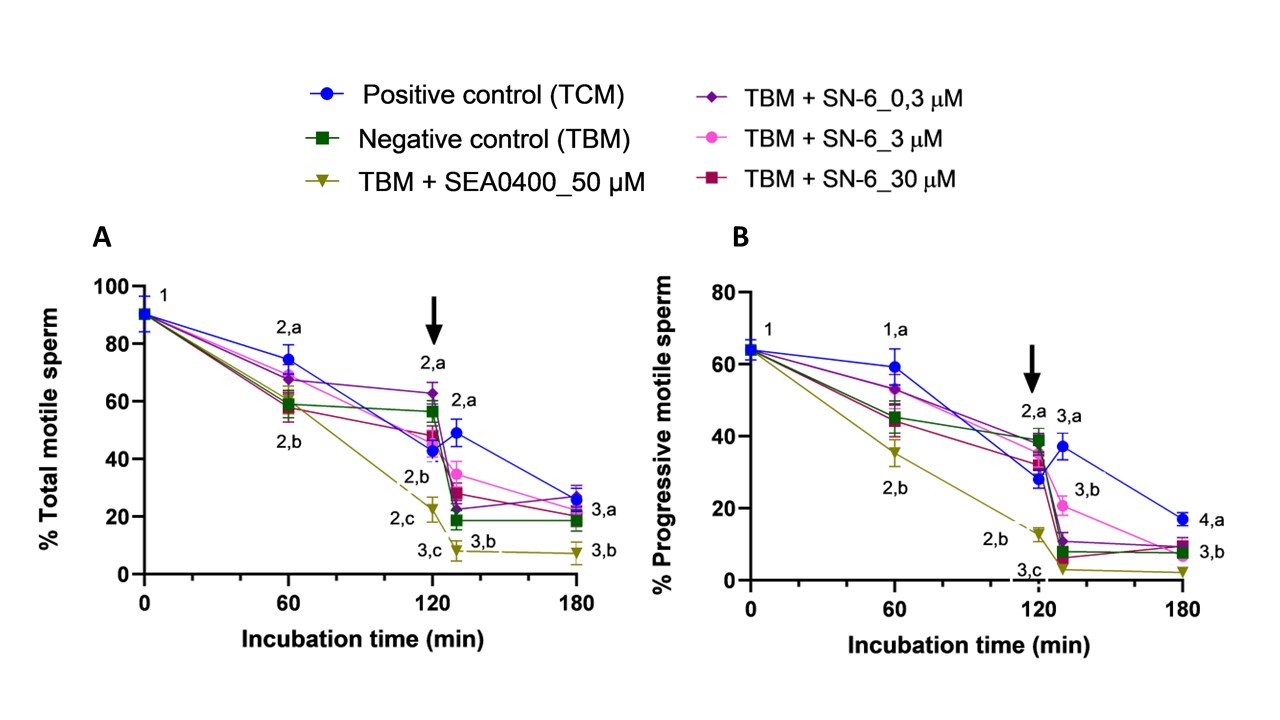


Suppl. Fig. 4**.** Effects of SEA0400 and SN-6 on sperm motility in non-capacitating conditions. Percentages of total (A) and progressively (B) motile sperm during in sperm samples incubated in non-capacitating medium (TBM) in the absence (negative control) or presence of NCX blocker (either 50 μM of SEA0400 or 0.3, 3 or 30 μM of SN-6). Samples incubated in capacitating medium were used as positive controls (TCM). Different superscript letters indicate significant differences between control and blocked samples within a single time point (*P* < 0.05). Different superscript numbers indicate significant differences between time points within a treatment (*P* < 0.05). The arrow indicates the addition of 10 μg/mL of progesterone at 120 min of incubation. Results are expressed as the mean ± SEM.


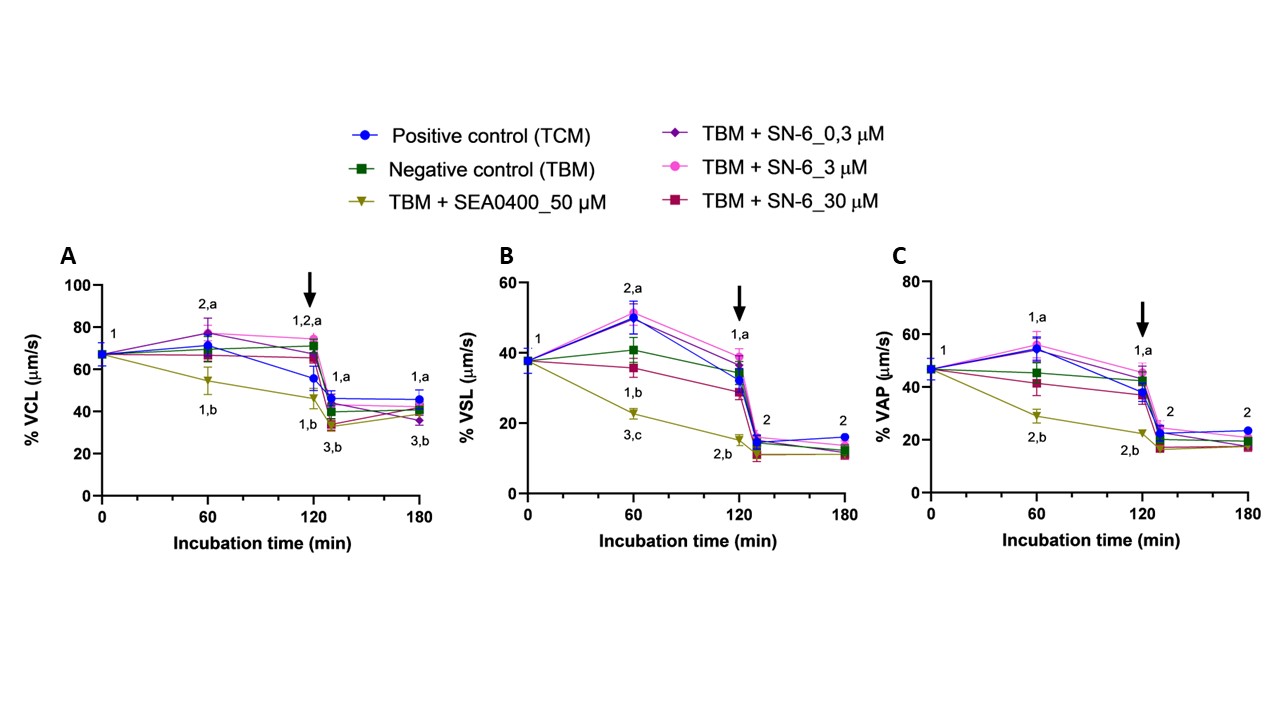


Suppl. Fig. 5**.** Effects of SEA0400 and SN-6 on sperm kinematics in non-capacitating conditions (I). Sperm velocity parameters of VCL (A), VSL (B), and VAP (C) in sperm samples incubated in non-capacitating medium (TBM) in the absence (negative control) or presence of NCX blocker (either 50 μM of SEA0400 or 0.3, 3 or 30 μM of SN-6). Samples incubated in capacitating medium were used as positive controls (TCM). Different superscript letters indicate significant differences between control and blocked samples within a single time point (*P* < 0.05). Different superscript numbers indicate significant differences between time points within a treatment (*P* < 0.05). The arrow indicates the addition of 10 μg/mL of progesterone at 120 min of incubation. Results are expressed as the mean ± SEM.


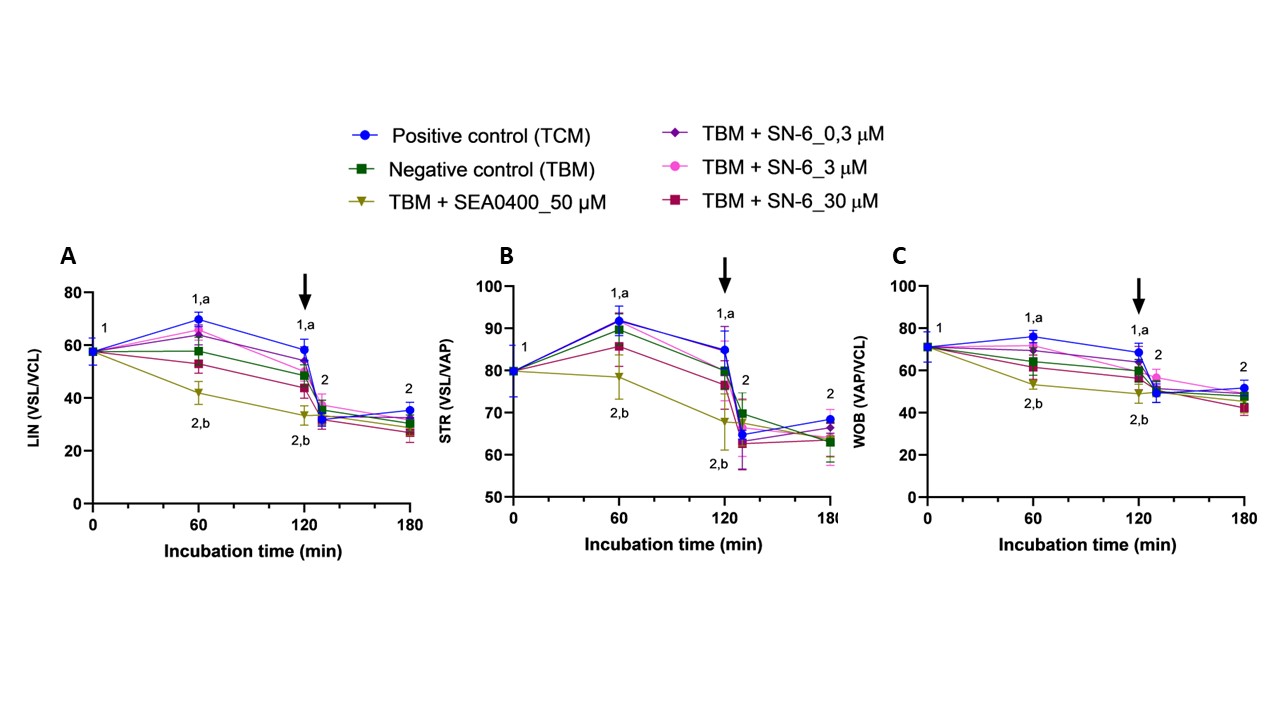


Suppl. Fig. 6**.** Effects of SEA0400 and SN-6 on sperm kinematics in non-capacitating conditions (II). Linearity (LIN, A), straightness (STR, B), and wobble (WOB, C) parameters in sperm samples incubated in non-capacitating medium (TBM) in the absence (negative control) or presence of NCX blocker (either 50 μM of SEA0400 or 0.3, 3 or 30 μM of SN-6). Samples incubated in capacitating medium were used as positive controls (TCM). Different superscript letters indicate significant differences between control and blocked samples within a single time point (*P* < 0.05). Different superscript numbers indicate significant differences between time points within a treatment (*P* < 0.05). The arrow indicates the addition of 10 μg/mL of progesterone at 120 min of incubation. Results are expressed as the mean ± SEM.


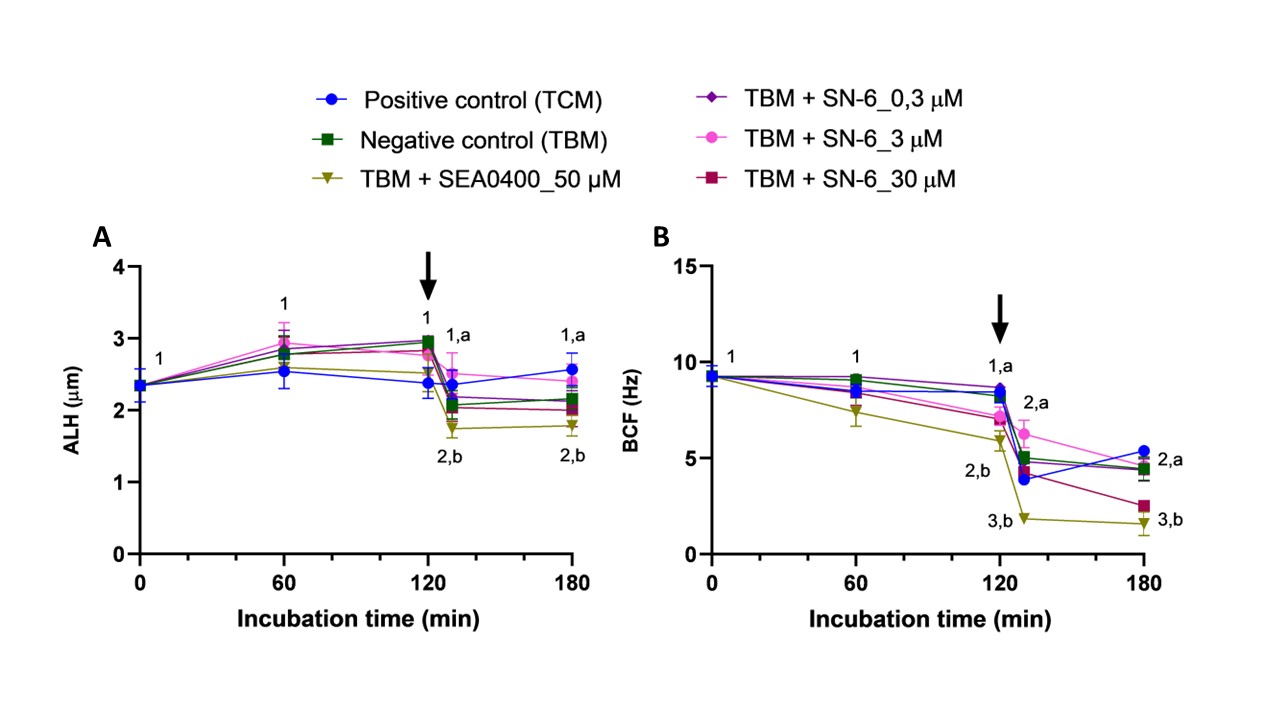


Suppl. Fig. 7**.** Effects of SEA0400 and SN-6 on sperm kinematics in non-capacitating conditions (III). Amplitude of lateral head displacement (ALH, A) and beat cross frequency (BCF, B) in sperm samples incubated in non-capacitating medium (TBM) in the absence (negative control) or presence of NCX blocker (either 50 μM of SEA0400 or 0.3, 3 or 30 μM of SN-6). Samples incubated in capacitating medium were used as positive controls (TCM). Different superscript letters indicate significant differences between control and blocked samples within a single time point (*P* < 0.05). Different superscript numbers indicate significant differences between time points within a treatment (*P* < 0.05). The arrow indicates the addition of 10 μg/mL of progesterone at 120 min of incubation. Results are expressed as the mean ± SEM.


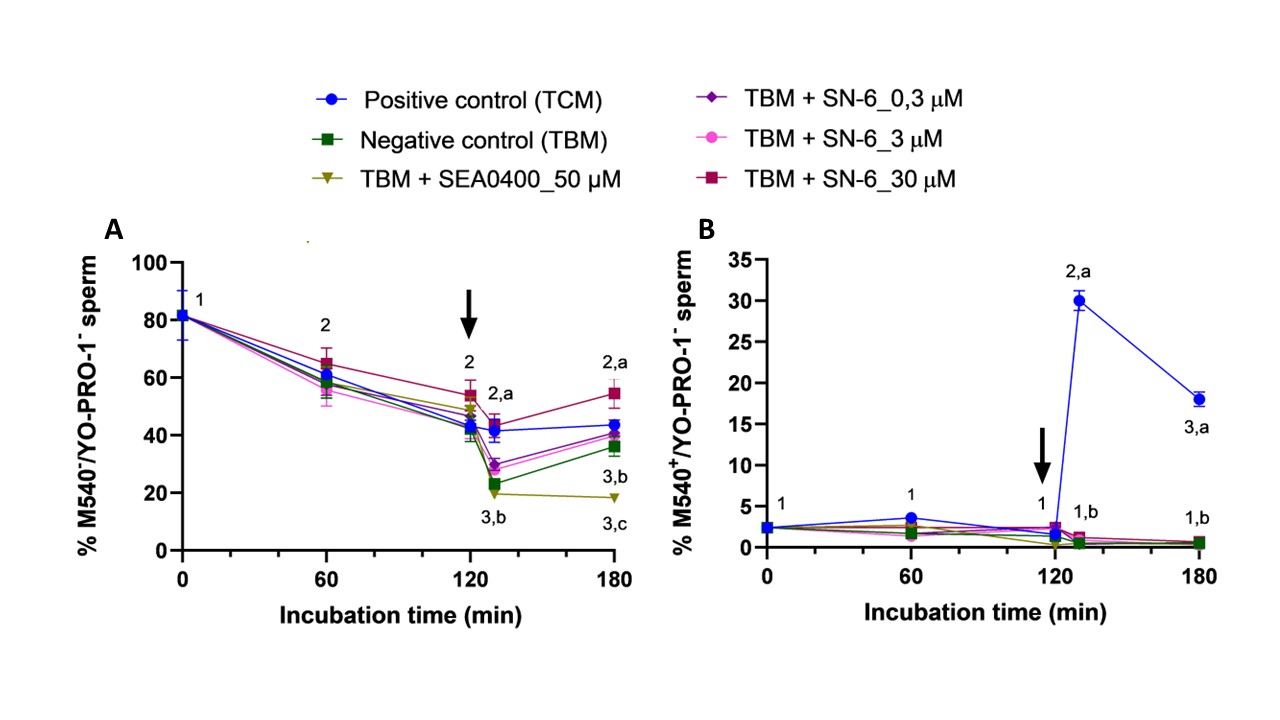


Suppl. Fig. 8**.** Effects of SEA0400 and SN-6 on lipid disorder of the plasma membrane in non-capacitating conditions. Percentages of viable sperm with low (M540^-^/YO-PRO-1^-^, A) and high (M540^+^/YO-PRO-1^-^, B) lipid disorder of the plasma membrane in sperm samples incubated in non-capacitating medium (TBM) in the absence (negative control) or presence of NCX blocker (either 50 μM of SEA0400 or 0.3, 3 or 30 μM of SN-6). Samples incubated in capacitating medium were used as positive controls (TCM). Different superscript letters indicate significant differences between control and blocked samples within a single time point (*P* < 0.05). Different superscript numbers indicate significant differences between time points within a treatment (*P* < 0.05). The arrow indicates the addition of 10 μg/mL of progesterone at 120 min of incubation. Results are expressed as the mean ± SEM.


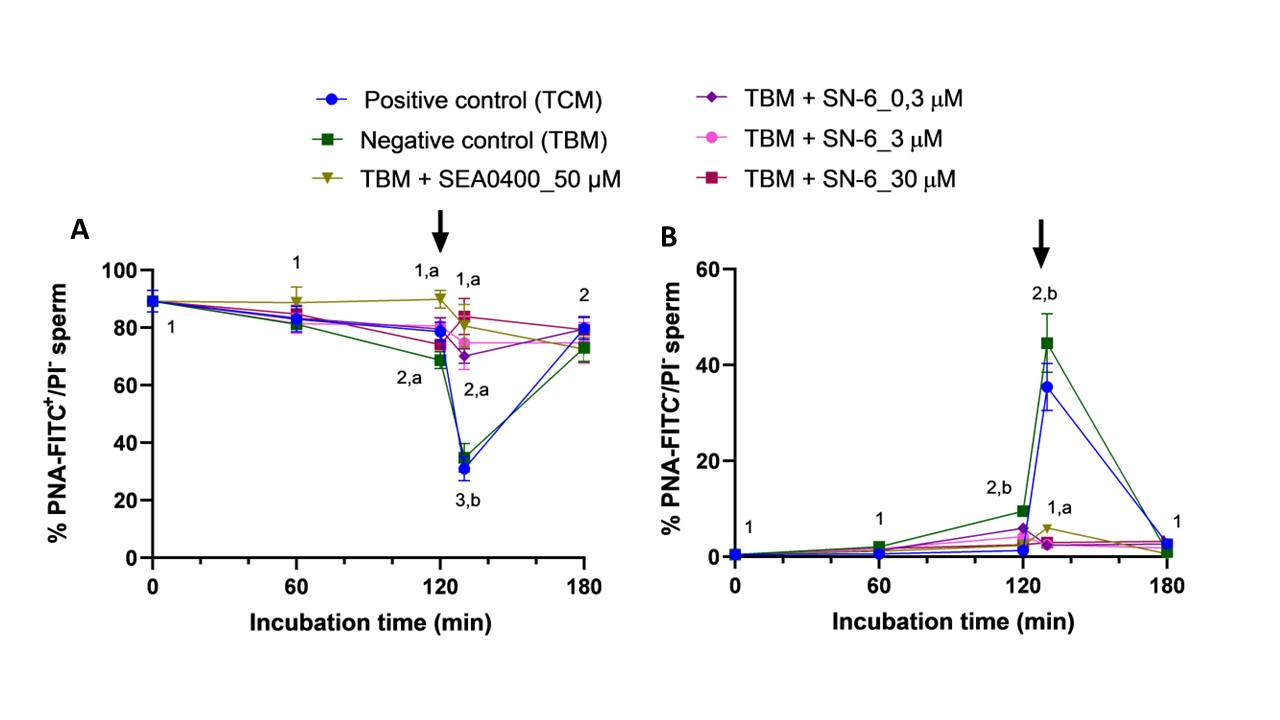
Suppl. Fig. 9**.** Effects of SEA0400 and SN-6 on acrosome integrity in non-capacitating conditions. Percentages of viable sperm with an intact acrosome (PNA-FITC+/EthD-1-, A) and with an exocytosed acrosome (PNA-FITC-/EthD-1-, B) in sperm samples incubated in non-capacitating medium (TBM) in the absence (negative control) or presence of NCX blocker (either 50 μM of SEA0400 or 0.3, 3 or 30 μM of SN-6). Samples incubated in capacitating medium were used as positive controls (TCM). Different superscript letters indicate significant differences between control and blocked samples within a single time point (*P* < 0.05). Different superscript numbers indicate significant differences between time points within a treatment (*P* < 0.05). The arrow indicates the addition of 10 μg/mL of progesterone at 120 min of incubation. Results are expressed as the mean ± SEM.


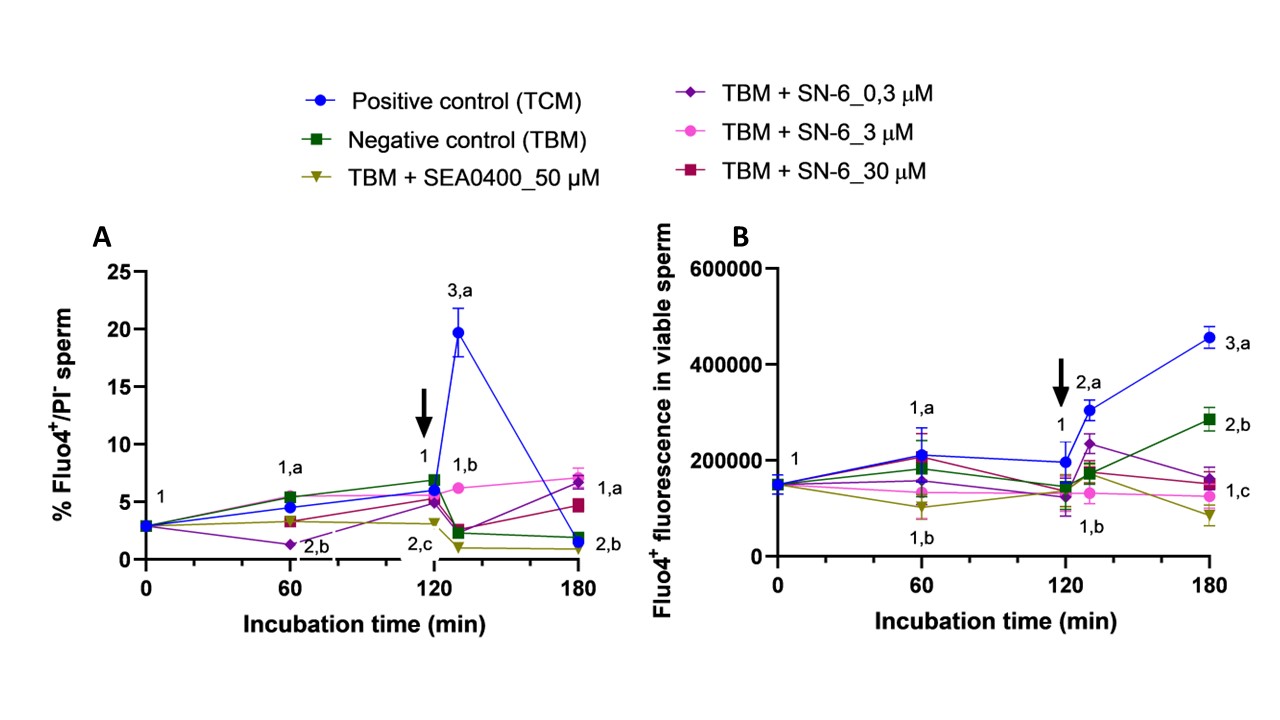


Suppl. Fig. 10**.** Effects of SEA0400 and SN-6 on intracellular Ca^2+^ levels in non-capacitating conditions. Percentages of viable sperm with high intracellular Ca^2+^ levels (Fluo4^+^/PI^-^, A) and fluorescence intensity of Fluo4^+^ in viable sperm (B) in sperm samples incubated in non-capacitating medium (TBM) in the absence (negative control) or presence of NCX blockers (either 50 μM of SEA0400 or 0.3, 3 or 30 μM of SN-6). Samples incubated in capacitating medium were used as positive controls (TCM). Different superscript letters indicate significant differences between control and blocked samples within a single time point (*P* < 0.05). Different superscript numbers indicate significant differences between time points within a treatment (*P* < 0.05). The arrow indicates the addition of 10 μg/mL of progesterone at 120 min of incubation. Results are expressed as the mean ± SEM.


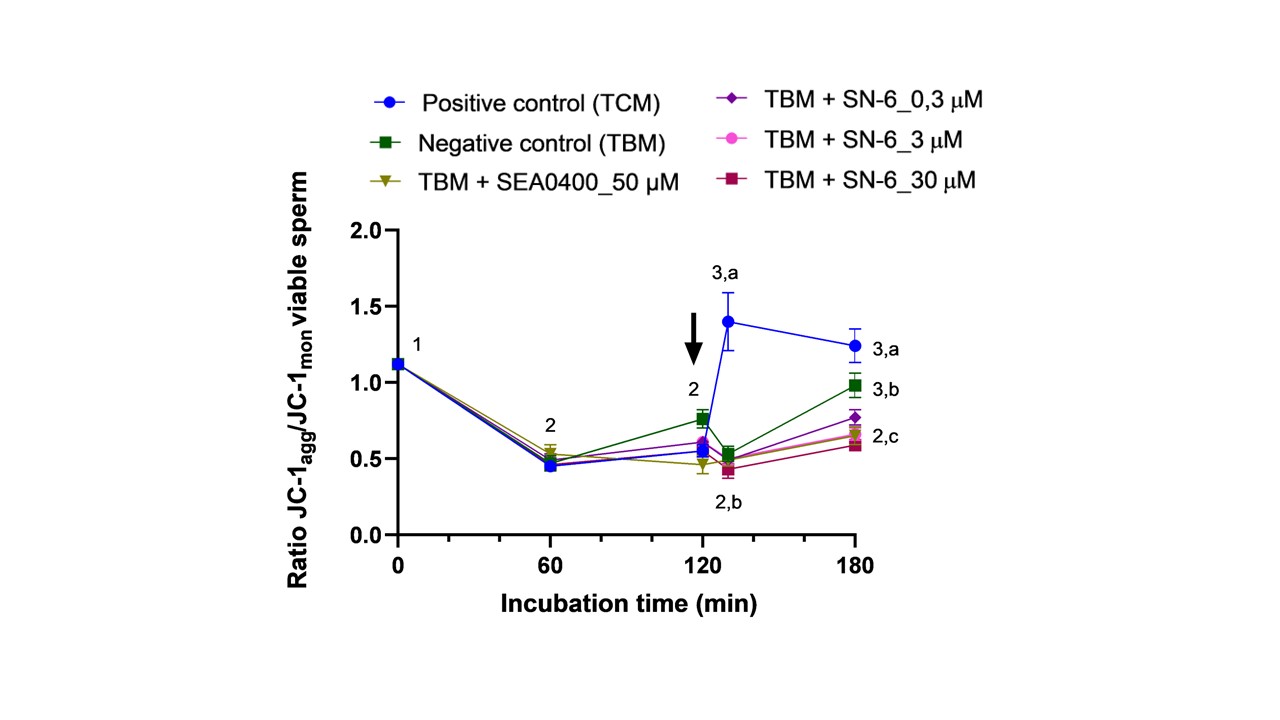


Suppl. Fig. 11**.** Effects of SEA0400 and SN-6 on mitochondrial membrane potential in non-capacitating conditions. Ratio between viable sperm (PI^-^) with high MMP (JC-1_agg_) and viable sperm with low MMP (JC1_mon_) in sperm samples incubated in non-capacitating medium (TBM) in the absence (negative control) or presence of NCX blocker (either 50 μM of SEA0400 or 0.3, 3 or 30 μM of SN-6). Samples incubated in capacitating medium were used as positive controls (TCM). Different superscript letters indicate significant differences between control and blocked samples within a single time point (*P* < 0.05). Different superscript numbers indicate significant differences between time points within a treatment (*P* < 0.05). The arrow indicates the addition of 10 μg/mL of progesterone at 120 min of incubation. Results are expressed as the mean ± SEM.


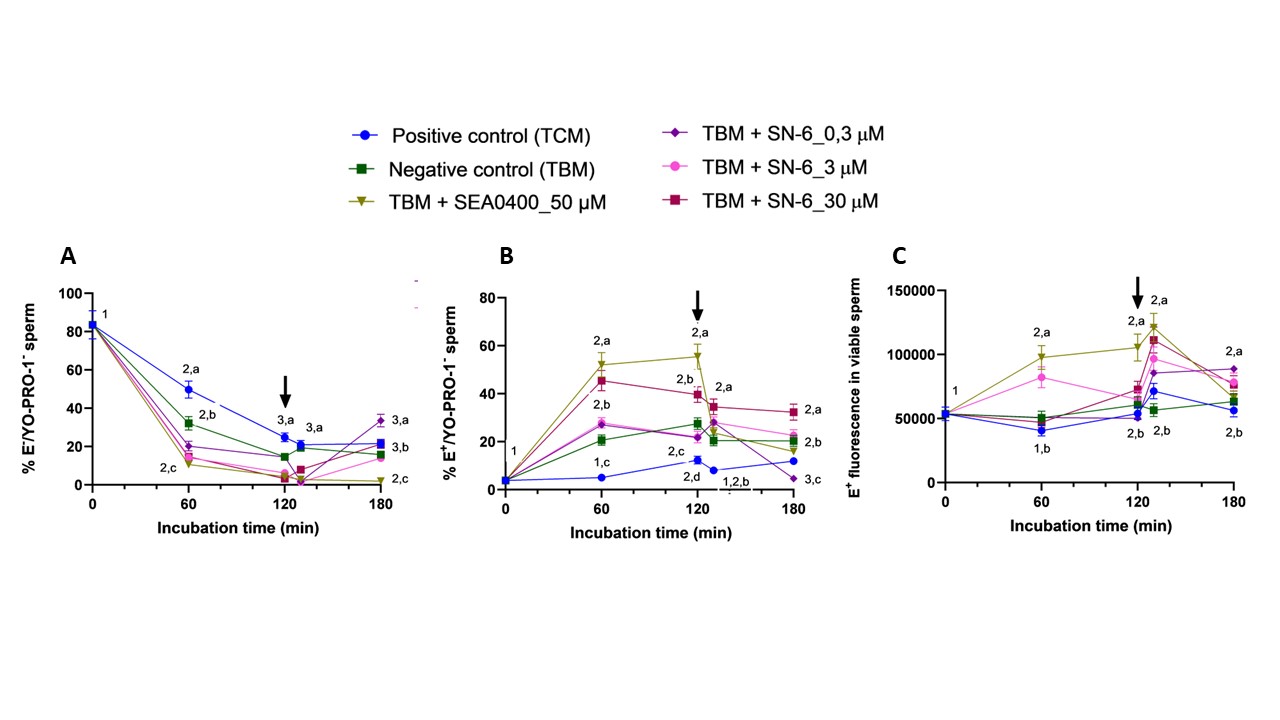


Suppl. Fig. 12**.** Effects of SEA0400 and SN-6 on ROS levels in non-capacitating conditions. Percentages of viable sperm with low (DCF^-^/PI^-^, A) and high (DCF^+^/PI^-^, B) ROS levels and fluorescence intensity of DCF^+^ in viable sperm (C) in sperm samples incubated in non-capacitating medium (TBM) in the absence (negative control) or presence of NCX blocker (either 50 μM of SEA0400 or 0.3, 3 or 30 μM of SN-6). Samples incubated in capacitating medium were used as positive controls (TCM). Different superscript letters indicate significant differences between control and blocked samples within a single time point (*P* < 0.05). Different superscript numbers indicate significant differences between time points within a treatment (*P* < 0.05). The arrow indicates the addition of 10 μg/mL of progesterone at 120 min of incubation. Results are expressed as the mean ± SEM.


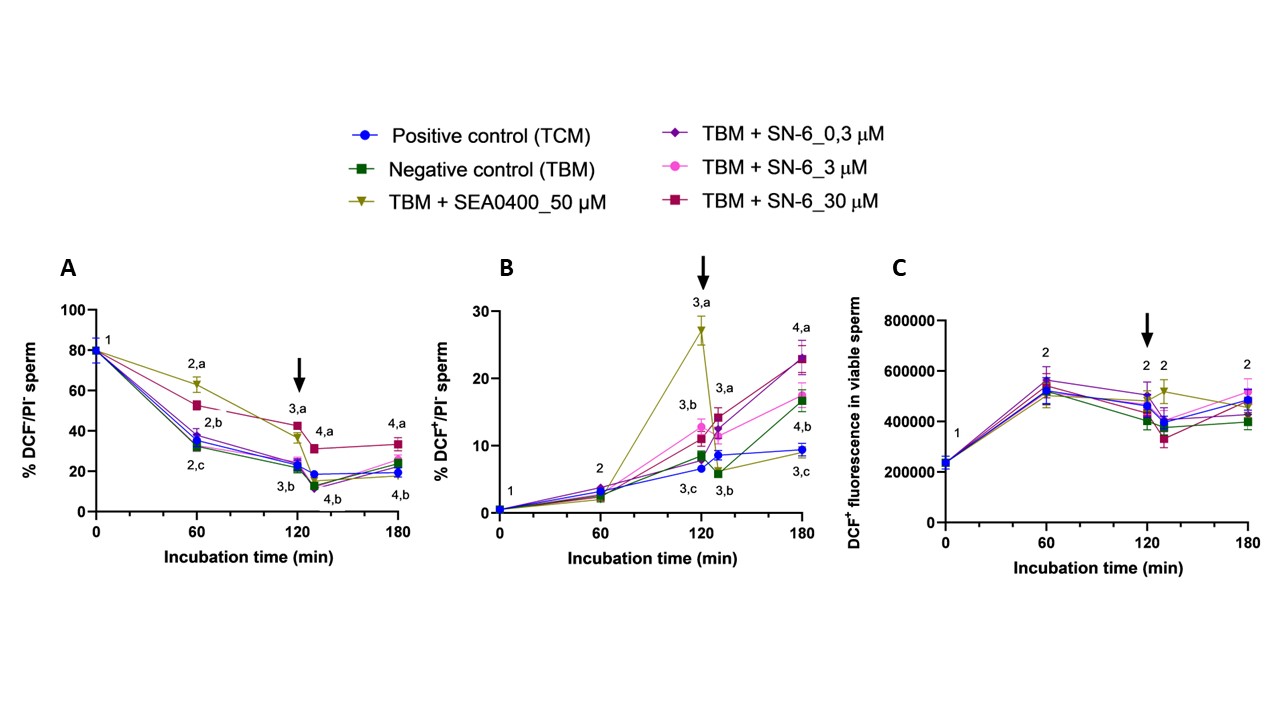


Suppl. Fig. 13**.** Effects of SEA0400 and SN-6 on superoxide levels in non-capacitating conditions. Percentages of viable sperm with low (E^-^/YO-PRO-1^-^, A) and high (E^+^/YO-PRO-1^-^, B) superoxide levels and fluorescence intensity of E^+^ in viable sperm (C) in sperm samples incubated in non-capacitating medium (TBM) in the absence (negative control) or presence of NCX blocker (either 50 μM of SEA0400 or 0.3, 3 or 30 μM of SN-6). Samples incubated in capacitating medium were used as positive controls (TCM). Different superscript letters indicate significant differences between control and blocked samples within a single time point (*P* < 0.05). Different superscript numbers indicate significant differences between time points within a treatment (*P* < 0.05). The arrow indicates the addition of 10 μg/mL of progesterone at 120 min of incubation. Results are expressed as the mean ± SEM.


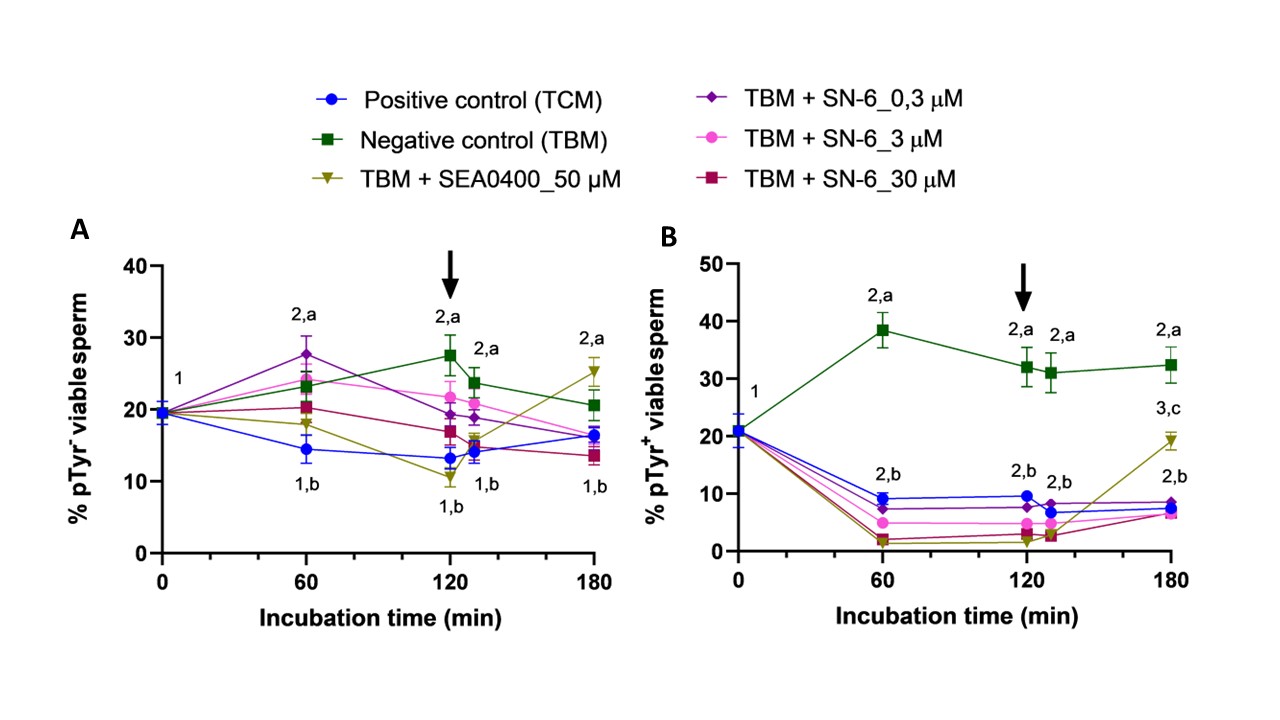


Suppl. Fig. 14**.** Effects of SEA0400 and SN-6 on phosphorylated tyrosine levels in non-capacitating conditions. Percentages of viable sperm with phosphorylated tyrosines (pTyr^+^, A) and fluorescence intensity of pTyr^+^ in viable sperm (B) in sperm samples incubated in non-capacitating medium (TBM) in the absence (negative control) or presence of NCX blocker (either 50 μM of SEA0400 or 0.3, 3 or 30 μM of SN-6). Samples incubated in capacitating medium were used as positive controls (TCM). Different superscript letters indicate significant differences between control and blocked samples within a single time point (*P* < 0.05). Different superscript numbers indicate significant differences between time points within a treatment (*P* < 0.05). The arrow indicates the addition of 10 μg/mL of progesterone at 120 min of incubation. Results are expressed as the mean ± SEM.
